# Nanostrategies for Therapeutic and Diagnostic Targeting of Gastrin-Releasing Peptide Receptor

**DOI:** 10.3390/ijms24043455

**Published:** 2023-02-09

**Authors:** Beata Paulina Rurarz, Małgorzata Bukowczyk, Natalia Gibka, Agnieszka Wanda Piastowska-Ciesielska, Urszula Karczmarczyk, Piotr Ulański

**Affiliations:** 1 Institute of Applied Radiation Chemistry, Faculty of Chemistry, Lodz University of Technology, Wroblewskiego 15, 93-590 Lodz, Poland; 2 Department of Cell Cultures and Genomic Analysis, Medical University of Lodz, Zeligowskiego 7/9, 90-752 Lodz, Poland; 3 National Centre for Nuclear Research, Radioisotope Centre POLATOM, Andrzeja Soltana 7, 05-400 Otwock, Poland

**Keywords:** gastrin-releasing peptide receptor, GRPR, bombesin receptor subtype 2, BB_2_, bombesin, targeted therapy, molecular imaging, theranostics, nanotechnology, nanoparticle, nanomedicine

## Abstract

Advances in nanomedicine bring the attention of researchers to the molecular targets that can play a major role in the development of novel therapeutic and diagnostic modalities for cancer management. The choice of a proper molecular target can decide the efficacy of the treatment and endorse the personalized medicine approach. Gastrin-releasing peptide receptor (GRPR) is a G-protein-coupled membrane receptor, well known to be overexpressed in numerous malignancies including pancreatic, prostate, breast, lung, colon, cervical, and gastrointestinal cancers. Therefore, many research groups express a deep interest in targeting GRPR with their nanoformulations. A broad spectrum of the GRPR ligands has been described in the literature, which allows tuning of the properties of the final formulation, particularly in the field of the ligand affinity to the receptor and internalization possibilities. Hereby, the recent advances in the field of applications of various nanoplatforms that are able to reach the GRPR-expressing cells are reviewed.

## 1. Introduction

Despite many efforts, cancer remains a major challenge for medicine professionals and scientists around the world. According to data published by the University of Oxford in cooperation with the Global Change Data Lab, cancer is the second leading cause of death worldwide [1]. Therefore, there is a constant need for new therapeutic and diagnostic modalities that can improve the current situation. Nanomedicine is believed to bring about change in the field of oncology and revolutionize current cancer management strategies. Markedly, targeted delivery of biologically active compounds is gaining momentum due to the high hopes they raise concerning improved therapeutic efficacy, convenient imaging and staging of malignancies, and minimized side effects on patients. Many molecular targets have been discovered to date, and most of the currently developed targeting strategies can be assigned to one of three general categories: uncontrolled cell proliferation targeting, angiogenesis-associated targeting, and specific tumor cells targeting [2]. Due to the fact that the majority of cancer-associated deaths are caused by five particular cancer types (tracheal, bronchus, and lung; colon and rectum; stomach; breast; pancreatic) [1], targeting the specific tumor cells is becoming particularly interesting. It can be successfully accomplished due to the specific receptors that are present or overexpressed in malignant, but not healthy cells. It is noteworthy that as low as threefold overexpression of a receptor on cancer cells already allows exploitation of the targeted delivery mechanism and successful delivery of the cargo [3]. Examples of receptors that were found to be overexpressed in malignant tissues are epidermal growth factor receptor (EGFR), folate receptor (FR), somatostatin receptors (SSTRs), or gastrin-releasing peptide receptor (GRPR). The latter was found to be overexpressed in most of the abovementioned cancers that are rising the death toll. Therefore, GRPR is attracting the particular attention of numerous research groups, and first formulations have already reached the clinical trials phase [4,5]. They focus on applications of GRPR ligands in combination with radioisotopes such as ^64^Cu and ^68^Ga for positron emission tomography (PET) in breast and prostate cancers [6,7]. Interest of the nuclear medicine community in GRPR targeting was also expressed by the launch of the Coordinated Research Project “Nanosized delivery systems for radiopharmaceuticals” by the International Atomic Energy Agency (IAEA) in 2014–2019 [8]. Researchers dealing with targeted nanomedicines and nuclear sciences have developed various strategies to exploit the potential of GRPR targeting with nanoformulations aiming at either cancer treatment, imaging, and diagnosis, or the combination of those two modalities. In their research, a myriad of targeting ligands leading to different biological responses upon the interaction with the receptor is accompanied by nanoparticles of diverse shape, origin, and composition. Acknowledging the significant interest in GRPR targeting, hereby, we have reviewed recent advances in the field of synthesis, characterization, and applications of various nanoplatforms which are able to reach the cells and tissues expressing gastrin-releasing peptide receptor, both in vitro and in vivo.

## 2. Gastrin-Releasing Peptide Receptor—Overview

**Figure 1:**
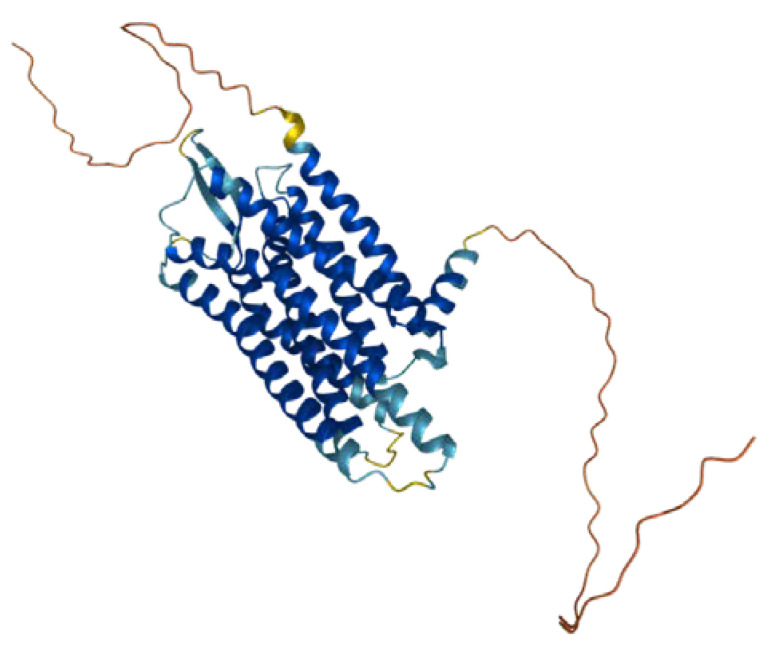
Structure of GRPR with seven transmembrane domains. Reprinted from ref. [13].

Gastrin-releasing peptide receptor (GRPR, also known as the BB2 receptor, Figure 1) is a member of the mammalian bombesin receptor family, comprising three distinct heptahelical receptors, having a wide range of physiological and pathophysiological effects. The human *GRPR* gene is located on the X chromosome, and the GRPR protein contains 384 amino acids of an estimated molecular weight of 43 kDa [9,10]. It is a member of the G-protein-coupled-receptors (GPCR) family, being a class of transmembrane proteins responsible for regulating a wide range of functions through ligand–receptor interactions. The interactions are based on the activation of heterotrimeric G protein subunits α and β/γ, which bind to target proteins, thus initiating respective cellular signaling pathways. The regions responsible for agonist binding have been found to be Gln120, Pro198, Arg287, and Ala307 [11], while the antagonist binding is possible due to the presence of Thr296, Phe301, and Ser304 [12]. An endogenous ligand of GRPR is gastric-releasing peptide (GRP).

Because GRPRs are known to be coupled with phospholipase C, they have the ability to induce the breakdown of phosphoinositides and the subsequent generation of diacylglycerol. This stimulates the mobilization of cellular calcium and the activation of protein kinases C (PKC), triggering a variety of cellular changes (e.g., membrane and cytosolic proteins or indirect regulation of cell proliferation). The stimulation of the receptor is also responsible for the activation of phospholipases β1 and β2, as well as tyrosine kinases or phosphorylation of some proteins, leading to processes such as the appearance of focal adhesion plaques or actin proliferation. The kinases belonging to the Src family are also rapidly activated, which has been found to be strongly influential on the invasion and growth of certain types of cancer. It is worth noting that chronic stimulation of GRPR receptors was found to strongly impact a variety of processes, such as internalization of receptor–ligand complexes, own-regulation of cell surface receptors, or receptor desensitization, all arising from a range of external or cellular stimuli [10].

**Figure 2:**
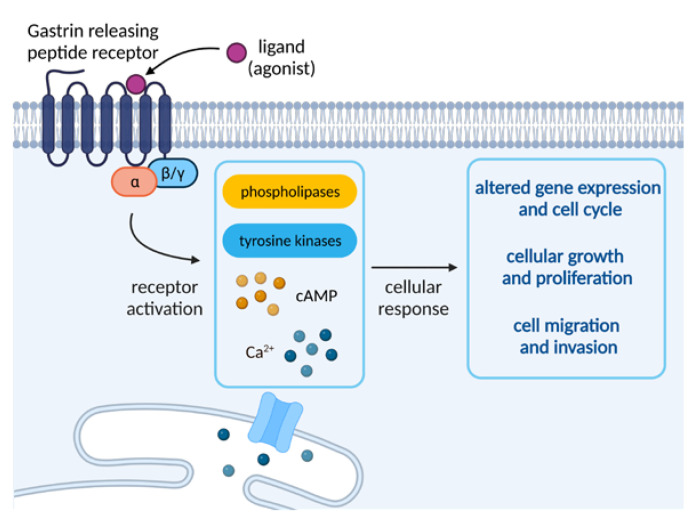
Overview of GRPR signaling. Created with BioRender.com.

GRPR activation entails the activation of phospholipases D and A2, as well as cyclic adenosine monophosphate (cAMP) increase in some tissues. It can also stimulate arresting translocation to the plasma membrane and has been found to enhance the growth of normal and neoplastic tissues. Moreover, the receptor activation has been found to stimulate the tumor cells to invade and migrate to healthy tissues via one of the Gα proteins or transesterification of the epidermal growth factor receptor (EGFR), which is said to influence the GRPR-stimulated DNA synthesis in tumor cells [10]. An overview of the molecular events upon activation of GRPR is depicted in Figure 2.

## 3. GRPR Targeting and Nanosystems

**Figure 3:**
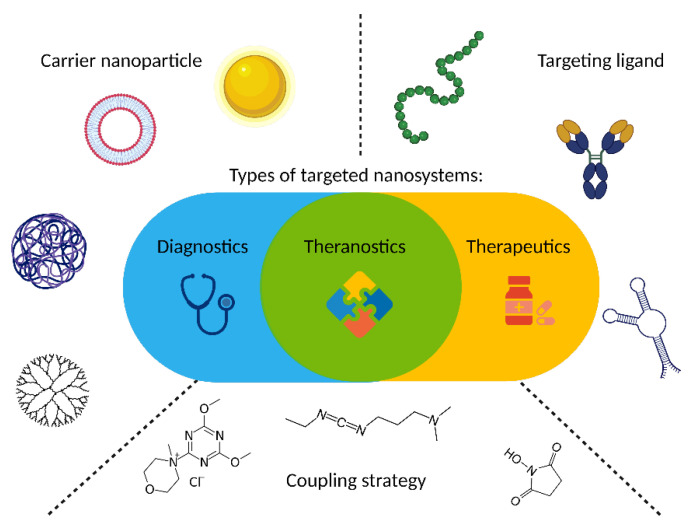
Targeted nanosystems may perform different roles, but, fundamentally, three major aspects, carrier nanoparticle, targeting ligand, and means of bringing them together, must be fine-tuned to yield successful formulation. Created with BioRender.com.

As GRPR is well acknowledged to be overexpressed in cancers, it attracts the attention of scientists dealing with novel delivery systems based on active targeting, where GRPR ligands are used as moieties able to lead to preferential accumulation of the nanoparticles in the sites of interest. These nanoparticles may act as drug delivery vehicles, so they deliver cytotoxic biological activity in the tumor site. On the other hand, detectable nanoparticles can be tracked in the human body using different imaging modalities, hence helping with cancer localization and staging. Finally, both these activities can be incorporated into one construct and act as theranostic, the idea derived from the personalized medicine approach, which connects oncological diagnostics and treatment at the same time. During the design of a targeted nanoformulation, fundamentally, three vital points need to be initially considered: the choice of the proper targeting ligand, the type of nanoparticles, and the means of bringing those together (Figure 3). These three aspects need to be fine-tuned to allow the successful development of nanosystems with anticipated properties and activity, particularly with respect to the desired mode of operation: therapeutic, diagnostic, or, combining both, theranostic (Figure 3).

There are certain trends that are noticeable in GRPR nanotargeting research. This receptor is overexpressed in numerous cancers, i.e., breast, colon, glioblastoma, head and neck squamous cell, lung, neuroblastoma, pancreatic, and prostate [3]. However, the vast majority (ca. 70%) of the published studies concerning the use of nanosystems focus on applications in prostate or breast cancer research. It seems coherent, though, with the public health data pointing out that those malignancies are (next to colon and rectum) in the top three of cancer prevalence by type worldwide [1]. However, when considering the type of nanoparticles exploited in the GRPR targeting research, almost half of the formulations are based on either gold nanostructures or liposomal/micellar vesicles, which are predominantly exploited in imaging and therapy, respectively. Those trends also seem well grounded, since, out of 25 clinically approved nanoformulations (U.S. Food and Drug Administration (FDA) and/or European Medicines Agency (EMA)), 10 were based on liposomes, proving the clinical potential of those structures [14]. Gold nanostructures may have not reached such clinical success yet, but the combination of relatively simple, cheap, and well-understood synthesis, beneficial biological half-life, convenient and stable modification due to universal gold–sulfur interactions, and a wide range of activity (photothermal therapy, contrasting agent for photoacoustic imaging, radiosensitization, etc.) make them appear very promising and worth exploring. 

### 3.1. Targeting Ligands

Ligands of GRPR can fall into one of two groups: agonists and antagonists. Agonists share the receptor activation sequence (Trp-Ala-Val-Gly-His-Leu-Met-NH_2_) with GRP and activate the GRPR signaling pathways, leading to downstream effects such as cell growth, proliferation, increased motility, and invasiveness. It was pointed out, though, that these effects are not desired in clinically viable formulations. Moreover, some bodies of evidence suggest that antagonists may perform better as targeting ligands [15]. Despite those flaws, surprisingly, most of the research published in the field of nanostrategies for targeting this receptor concerns the use of agonists. One of the possible reasons might be that internalization of the nanoparticle delivering the cargo, together with the receptor upon its activation, may be beneficial for some drug delivery applications and is convenient to track in vitro. However, actual reasons for this state of the matter remain to be elucidated and may be much more trivial, e.g., easy access and broad awareness of bombesin (BBN), the best known GRPR ligand, which happens to be an agonist.

**Figure 4:**
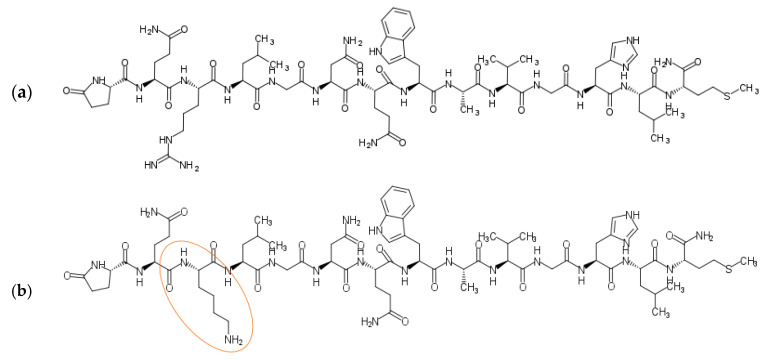
Structures of commercially available bombesin peptides: (**a**) BBN (Pyr-Gln-Arg-Leu-Gly-Asn-Gln-Trp-Ala-Val-Gly-His-Leu-Met-NH_2_); (**b**) [Lys3]BBN (Pyr-Gln-Lys-Leu-Gly-Asn-Gln-Trp-Ala-Val-Gly-His-Leu-Met-NH_2_). Lysine included in the modified BBN sequence is labeled orange. Sketched with MarvinSketch 22.13 (ChemAxon Ltd., Budapest, Hungary).

The wide range of agonistic ligands has been shown in the literature. The relatively easiest approach is to use the commercially available native sequence of bombesin [16] (Figure 4a). Kulhari and co-workers exploited it in combination with biodegradable poly(lactic-co-glycolic acid) (PLGA) nanoparticles to successfully target both breast and prostate cancer cells and improve docetaxel (DTX) anticancer activity [17,18,19]. The same ligand was also used to target the [^64^Cu]CuS nanoparticles toward prostate cancer by Cai et al. [20]. Interestingly, using native bombesin, Du, and Li investigated if the synthetic strategy, namely pre/post-functionalization of nanostructured lipid carriers, affects the performance of the nanosystem for targeted lung cancer combination therapy [21]. 

Variations on the ligand structures start with exchanging single amino acids to engineer the sequence towards facilitated conjugation, for example, replacing the third amino acid from the native sequence with lysin [22,23,24], therefore providing additional -NH_2_ groups—such ligands are also commercially available [16] (Figure 4b).

Nevertheless, much more sophisticated structures were also developed. One of the most prominent alterations in the bombesin architecture is to shorten the peptide chain down to an essential receptor binding sequence, Trp-Ala-Val-Gly-His-Leu-Met-NH_2_, which can lower the price of the custom peptide synthesis. These short peptide chains can also be further engineered to provide convenient means of condensation with nanoparticles [25,26,27], which will be discussed in more detail in the next subchapter. Frequently, the ligand is combined with poly(ethylene glycol) (PEG) to improve the pharmacokinetics of the construct and minimize the reticuloendothelial system uptake upon in vivo administration [28,29].

Adding extra functionality to the structure represents a very attractive strategy for bombesin structure alterations. An example of such modifications is introducing the radioisotope chelation functionality to the ligand. Two main chelators used in such research are 1,4,7,10-tetraazacyclododecane-1,4,7,10-tetraacetic acid (DOTA) and hydrazinonicotinic acid (HYNIC), and the isotopes used are ^177^Lu and ^99m^Tc, respectively [30,31]. Another interesting idea is to combine the bombesin binding motif with another peptide, creating heteromultimeric ligands, which can improve the construct targeting ability (e.g., RGD sequence against integrin α_υ_β_3_ [32,33], prostate-specific membrane antigen (PSMA)-inhibiting peptides for targeting of prostate cancer) or cellular penetration (e.g., Tat(49–57), HIV-1-derived sequence [34,35]). 

Regarding the antagonistic ligands, GRPR antagonists can be categorized into six classes, out of which, five are peptides/peptoids, while the last class comprises low-molecular flavone derivatives [10]. Classes 2, 3, and 4 (in contrast to classes 1 and 5) are based on the modifications of the binding region of BBN. Class 2 is D-Phe12 analogs; class 3 focuses on modifications in positions 13–14 (or 26–27 when considering GRP sequence), while class 4 comprises analogs devoid of Met14 (desMet14). Among all the papers hereby reviewed, only six deal with the nanosystems exploiting antagonist ligands. Lahooti and colleagues used a class 2 antagonist with D-Phe12 substitution to successfully target their ultrasmall paramagnetic iron oxide nanoparticles (USPION) to GRPR-positive breast cancer xenograft in vivo [36]. Particular attention was given to class 3 antagonist D-Phe-Gln-Trp-Ala-Val- NMeGly-His-Sta-Leu-NH_2_ (BBN-AA1) by the group of Accardo. They successfully incorporated this sequence into amphiphilic derivative monomer, which was used to form liposomes loaded with doxorubicin (DOX) [37]. The same monomer was also used to form sterically stabilized micelles as a targeted carrier of poorly water-soluble anticancer drug—gold(III) dithiocarbamate [38]. The BBN-AA1 sequence with some spacers and maleimide-reactive cysteine was used to obtain the kit for targeted DOX-loaded liposomes, compliant with Good Manufacturing Practice (GMP), with satisfactory stability [39]. Li and co-workers exploited another class 3 antagonist sequence: [D-Phe6-Sta13-Leu14-NH_2_]bombesin(6–14) [40]. In their research, this sequence was further modified with Alexa Fluor 750 to obtain a carrier system for molecular imaging of oral squamous cell carcinoma. Tagliviani and co-workers exploited Demobesin-1—a GRPR peptide antagonist that falls into the class 4 analogs [41]. They have shown that, upon adding a trioxatridecan-succinamic acid spacer, Demobesin-1 provided successful cell recognition functionality to the polyoxometalate clusters.

Accardo et al. analyzed the performance of seven different GRPR antagonists based on DOTA and BBN derivatives coupled with PEG—formulations with a very simple design but offering an insight into the mechanisms by which they interact with GRPRs and, hence, provide varying targeting yield and diagnosis options [29]. From their systematic analysis, they concluded that most of the examined BBN derivatives serve equally well for targeting purposes (which was confirmed with gamma camera recordings), but those with NMeGly11 and Sta13-Leu14 would be most suitable for imaging in vivo, since their plasma half-lives are over 15 days, and the latter also provides a favorable absorption mostly into the tumor tissues and no other organs of the body. 

As mentioned above, GRPR is the mammalian bombesin receptor family member, comprising three distinct receptors. Basically, each of these receptors is expressed in different neoplastic conditions and has its own set of ligands, however, the universal binding sequence was discovered (D-Tyr6-Gln7-Trp8-Ala9-Val10-βAla11-His12-Phe13-Nle14), sometimes referred to as pan-bombesin. This finding is particularly attractive because with such a ligand it is possible to target tumors expressing various bombesin receptors patterns while improving the construct’s tumor-targeting efficiency and applicability. Heidari and co-workers used a peptide based on a universal binding sequence (Lys-Gly-Gly-Cys-Asp-Phe-Gln-Trp-Ala-Val-bAla-His-Phe-Nle) to target breast cancer in vitro and in vivo with gold nanorods for photothermal therapy [42] as well as imaging [43], and they pointed out its several advantages in comparison to native bombesin sequence, such as overcoming the issues related to receptor heterogeneity in tumors and ameliorating renal clearance due to the negatively charged hydrophilic aspartic acid residue. Similarly, Salouti et al. [44] and Jafari et al. [45] proposed the application of this peptide in breast cancer imaging using gold and superparamagnetic iron oxide nanoparticles, respectively. Pan-bombesin was also shown to improve prostate cancer imaging [46,47].

### 3.2. Ligand Incorporation

**Figure 5:**
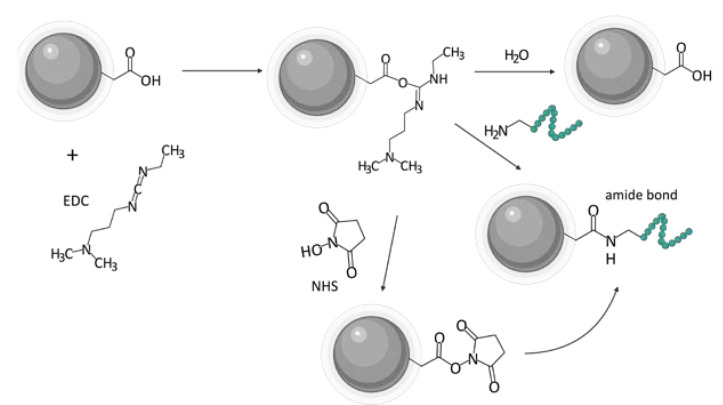
Scheme of the EDC/NHS chemistry of ligand incorporation on the example of carboxylic group tethered nanoparticle. Created with BioRender.com. Chemical structures sketched with MarvinSketch 22.13 (ChemAxon Ltd., Budapest, Hungary).

One of the biggest challenges in the synthesis of particles targeting GRPR or any other receptor is the incorporation of the targeting ligand in the structure of the vehicle. Various approaches can be found in the literature. A state-of-the-art technique based on the formation of an amide linkage between the ligand and the nanoparticle is most commonly used. Chemistry of 1-ethyl-3-(3-dimethyl aminopropyl)carbodiimide (EDC, EDAC) in combination with N-hydroxysuccinimide (NHS) [24] or sulfo-N-hydroxysuccinimide (sulfo-NHS) [43,48] is most commonly used to create the bond. Usually, this amide is formed between the N-terminus of the peptide ligands and carboxyls on the nanoparticles (Figure 5). Tang and co-workers successfully decorated their poly(acrylic acid)-functionalized lanthanide nanoparticles with BBN analog using EDC/sulfo-NHS chemistry [49]. It was even shown to be feasible to prepare ready-to-use kits with, e.g., stable NHS esters. EDC/NHS reactions are usually led in water, which is beneficial in terms of biocompatibility and purification procedures. However, the reaction, for instance, in tetrahydrofuran (THF) or dimethylformamide (DMF) is also possible, as shown by Du and Li [21] and Mansour et al. [50], respectively.

Nevertheless, with no carboxylic groups available on the surface of the nanoparticle, or no amine group available in the ligand, the other way around can be equally efficient [20,51]. Poly(amidoamine) (PAMAM) dendrimers were conjugated with DOTA-modified BBN using the amine group on the dendrimer and carboxylic group of the DOTA moiety [52,53]. Hajiramezanali and co-workers used this approach to exploit amine groups on chitosan and conjugate succinylated BBN derivative [54]. However, organic solvents and water-insoluble substrates are also used, such as 2-(1H-7-azabenzotriazol-1-yl)-1.1.3.3-tetramethyluroniumhexafluorophosphate) (HATU) [53,55,56] or N,N′-dicyclohexyl carbodiimide (DCC) [41,57] in DMF with the addition of N,N-diisopropylethylamine (DIPEA) [53]. In addition, click chemistry such as thiol-maleimide [39,58,59] or copper-catalyzed azide-alkyne cycloaddition was useful [46,47,60,61]. Dash and colleagues used cysteine groups incorporated in the peptide structure to click the GRPR-binding peptide with maleimide-containing PEGylated magnetic reduced graphene oxide [62].

**Figure 6:**
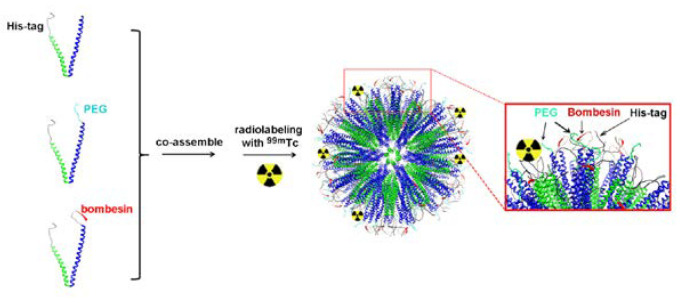
GRPR binding motif can be incorporated in the structure of the building blocks of the nanostructure prior to its assembly. Adapted from ref. [66].

In some cases, the ligand can be incorporated into the structure of particle-forming substrates before the particle is formed [63]. Accardo et al. have used solid-phase peptide synthesis with Fmoc/tBu chemistry to synthesize the ligand peptide [DOTA-bAla]BBN(7–14) and combine it with the amphiphilic monomers [64]. Subsequently, these monomers were cleaved from the Rink amide resin, purified, and assembled into targeted supramolecular aggregates. A similar approach was adopted by Accardo and co-workers to prepare DOX-loaded targeted liposomes [37]. Kanazawa et al. used this approach to combine bombesin with stearic acid directly in solid-phase peptide synthesis, and they used it for polymeric micelles [65]. Yang and co-workers incorporated the GRPR binding motif of BBN into the structure of their engineered peptides able to form a stable coiled-coil nanostructure [66]. An interesting approach was also presented by Zhang and colleagues—they have genetically engineered *Escherichia coli* bacteria to express elastin-like peptides capable of coassembly into micelles [67]. One of the peptides was engineered to contain a GRP-derived binding sequence (Figure 6).

In some instances, the application of a linker is necessary due to the configuration of functional groups present in both ligand and particle; both homo- and heterobifunctional linkers are available. For example, Achilli et al. used glutaraldehyde as a homobifunctional amine-reactive crosslinker to functionalize human serum albumin (HSA) protein corona of biohybrid gold nanoparticles with [Lys1-Lys3(DOTA)]BBN [68]. Kim et al. tethered bombesin ligand to amine-modified poly(ethylene glycol) block of amphiphilic copolymer using a heterobifunctional linker, succinimidyl 4-(N-maleimidomethyl)cyclohexane-1-carboxylate (SMCC) [69]. Montet et al. have used amine- and thiol-reactive succinimidyl iodoacetic acid to ligate the BBN derivative with amine-containing iron oxide nanoparticles [70]. Lee and co-workers used N-succinimidyl 3-(2-pyridyldithio)propionate (SPDP) to ligate cysteine-terminated derivative with glycol chitosan nanoparticles [71].

**Figure 7:**
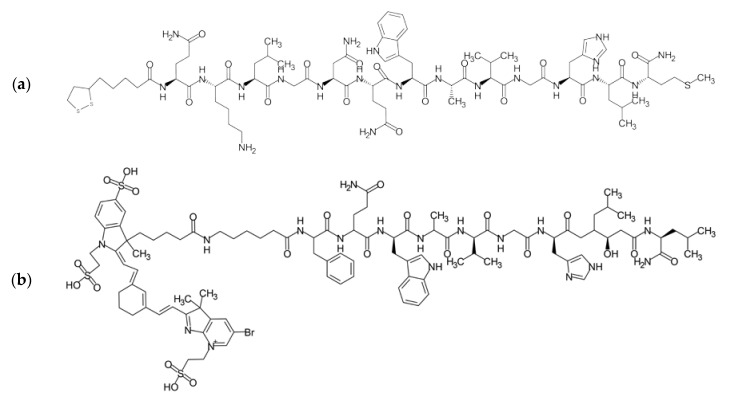
Structures of bombesin derivatives: (**a**) modified with thioctic acid for binding with gold nanoparticles [72]; (**b**) AF750-6Ahx-Sta-BBN for biding with nanographene oxide via hydrogen and π–π interactions [40]. Sketched with MarvinSketch 22.13 (ChemAxon Ltd., Budapest, Hungary).

In addition, noncovalent bonding may be useful in this context (Figure 7). Successful attachment of the BBN analog to gold nanoparticles can be achieved by exploiting the interaction between nitrogen atoms in free amine groups and the gold surface [23,44]. Ocampo-Garcia et al. have developed a shelf-storage stable kit based on gold nanoparticles operating on this principle [22]. On the other hand, Chanda and co-workers used the thioctic-acid-modified bombesin derivative to exploit thiol–gold interactions for stable functionalization (stable even in a reducing environment of dithiothreitol) [72]. Hosta-Rigau et al. used additional cysteine to provide thiol for interaction with gold [73]. Combining functional linkers such as N-succinimidyl 3-(2-pyridyldithio)propionate (SPDP) and gold–sulfur interactions can be an interesting alternative for gold nanoparticles [26,71]. Li and colleagues exploited hydrogen bonds and π–π stacking to functionalize nano-graphene oxide with a Sta-BBN ligand [40]. Interestingly, Trujillo-Benítez and co-workers used the DOTA moiety incorporated in the BBN derivative structure to achieve stable chelation of Sm atoms and, therefore, ligation of the ligand with metal oxide nanoparticles [33]. Young et al. have used one of the strongest known noncovalent interaction systems, biotin–streptavidin, to bind biotinylated bombesin to quantum dots decorated with streptavidin, resulting in efficient GRPR-selective fluorescent label operating in vivo [74]. 

**Figure 8:**
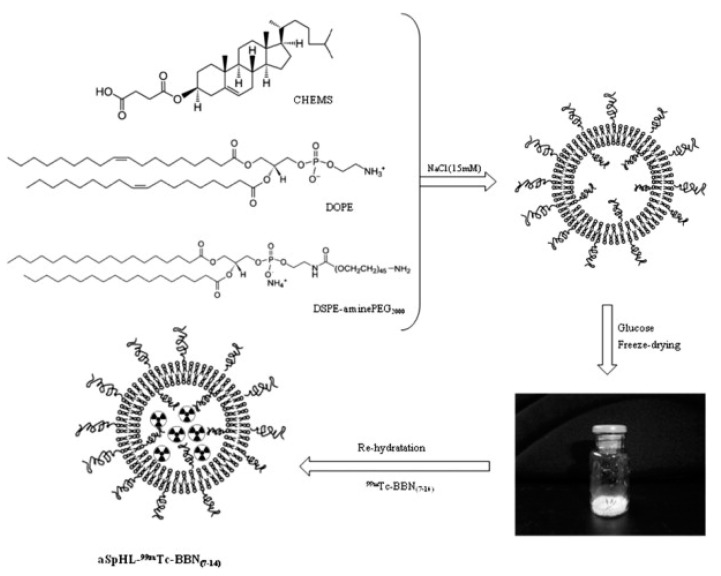
Technetium-99m labeled BBN(7–14) ligand can be incorporated in the liposome by encapsulating it inside the vesicle. CHEMS—cholesteryl hemisuccinate; DOPE—dioleoylphosphatidylethanolamine. Reprinted with permission from ref. [75]. Copyright 2022, Elsevier.

Finally, a ligand can also be protected inside the nanoparticle. De Barros et al. postulate that, due to natural plasma and tissue peptidases, peptides should be protected inside the nanocarrier; therefore, they have encapsulated radiolabeled bombesin derivatives inside the long-circulating pH-sensitive liposomes (Figure 8). This approach led to the successful targeting of GRPR-positive breast cancer and Ehrlich tumor cells [75,76,77].

## 4. Targeted Delivery of Biologically-Active Compounds

Table 1 provides examples of contemporary (ca. 2010 to date) advances in the research concerning targeted delivery applications of nanocarriers functionalized with GRPR-avid moieties. 

### 4.1. Micelles, Liposomes, and Other Self-Assembled Structures

**Figure 9:**
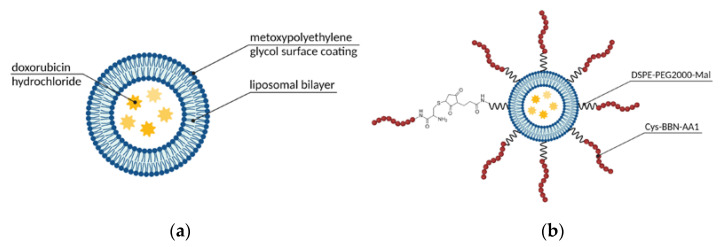
Structures of (**a**) Doxil (PEGylated liposomal doxorubicin) (inspired by ref. [83]) and (**b**) Doxil conjugated with BBN employing DSPE-PEG2000-maleimide (inspired by ref. [39]). Created with BioRender.com.

One of the most commonly used vehicles in drug delivery is supramolecular assemblies with a micellar or liposomal arrangement, following the patterns we can find in natural systems such as cell membranes. These materials offer a number of advantages, namely biocompatibility, a broad spectrum of functionalization possibilities, precisely controlled size, and convenient, still robust synthesis [64]. Therefore, it is no surprise that the first FDA-approved nanotechnology-based drug was a liposomal formulation. Since Doxil, liposomal DOX (Figure 9a), was launched, researchers have continued the dynamic exploration of the field of liposomal and micellar formulations to search for further improvements, one of those being active targeting of GRPR-overexpressing tumors, such as prostate cancer [39] or small-cell lung cancer [59]. Much effort was shown in this area by Accardo and co-workers. They have examined multiple targeting ligand alternatives (as described above in Section 3.1) to choose the one that performs the best and used it in multiple studies regarding the development of self-assembled structures. Their most recent communication published in this matter complements their efforts in a form of the kit for industry-friendly prepared liposomal DOX targeted with the engineered amphiphilic ligand containing a bombesin active sequence and phospholipidic hydrocarbon chain (Figure 9b) [39]. Their kit contains three vials: (a) liposomal doxorubicin, (b) BBN-reactive freeze-dried phospholipid, and (c) bombesin analog. The conjugate is efficiently formed in situ, with no loss of DOX or size alterations, and was shown to significantly inhibit tumor growth in comparison to non-targeted liposomal DOX. 

Among the methods enabling liposome preparation, one can mention, e.g., the solvent evaporation method with subsequent film rehydration or simple emulsification and solvent evaporation. However, more sophisticated methods are also developed—Maqbool et al. used supercritical carbon dioxide technology to produce their albendazole-loaded liposomes [78]. They have successfully synthesized structures of ca. 160 nm size. Although functionalization with palmitic acid-anchored BBN(6–14) led to a slight increase in size, BBN-boosted specific uptake significantly improved liposomes cytotoxicity in the in vitro prostate cancer model in comparison to free drug.

Micellar vesicles also can be obtained by simple procedures. Kanazawa et al. prepared poly(ethylene glycol)-polycaprolactone (PEG-PCL) block polymeric micelles by creating water-in-oil (w/o) emulsions based on ultrapure water and drug solutions in organic solvents [65]. The aim was to design a glioma-targeting nanocarrier with the ability to cross the blood–brain barrier and deliver the therapeutic of choice to the GRPR-overexpressing cells of the tumor. Therefore, micelles were functionalized with stearoyl-modified BBN and Tat(49–57), as well as loaded either with coumarin or camptothecin (CPT). Both formulations were tested as safe and alone had no effect on the cells. The coumarin-loaded BBN-conjugated nanocarriers showed an improved C6 cell uptake over PEG-PCL-Tat(49–57) (17 vs. 10%), and the researchers established that their absorption into the tumor might occur through macropinocytosis by Tat(49–57) and clathrin-mediated endocytosis by BBN, making bombesin an important modulator of the intratumoral uptake. In vivo studies in orthotopic glioma-grafted rats confirmed in vitro findings and showed an enhanced coumarin uptake in cancerous tissue as well as reduced internalization into healthy tissues. In the case of CPT, the release of the drug from BBN-conjugated nanocarriers was greater as compared to pure PEG-PCL-Tat(49–57), but the difference was not significant. Nonetheless, the targeting transporters performed noticeably better in cytotoxicity studies in C6 glioma cells as well as prolonged the life of tumor-bearing rats. Their post-intranasal administration lifespan was extended from 17.8 days (for untreated rats) to 20.3 days, 30.8 days, and 42.2 days for pure CPT, CPT-loaded PEG-PCL-Tat(49–57), and BBN/PEG-PCL-Tat(49–57) respectively, with 2/5 of the population surviving over 120 days in the case of GRPR-targeting nanoformulations. What is also important is that the micelle encapsulation of the drug made it less toxic to other bodily tissues and organs. It was revealed that there was no weight loss post-intranasal administration, in contrast to a drastic drop in body weight in the case of pure CPT solution. 

Fine-tuning of composition can relatively easily confer desired features to the assemblies, such as high drug-loading capacity, controlled release profile, prolonged circulation time, or slow bloodstream clearance. Because the hydrophobic compartments of liposomes and micelles can host poorly water-soluble compounds, their bioavailability and convenience of use can be significantly improved by carriers’ applications. Ringhieri and colleagues used sterically-stabilized micelles based on 1,2-stearoyl-sn-glycerol-3-phosphoethanolamine-N-[amino(polyethylene glycol)-2000] (DSPE-PEG2000) to encapsulate highly toxic anticancer drug with low water solubility Au(III)-dithiocarbamate complex (AuL12) [38]. Small micelles (~20 nm) were obtained, and the drug was successfully loaded into the micelles. Neither loading nor targeting moiety incorporation affected the structure size or the optimization of composition, however, the incorporation of egg L-α-phosphatidylcholine (PC) or 1,2-dioleoyl-sn-glycerol-3-phosphocholine (DOPC) increased the loading capacity. The drug was shown to be stable in the liposomes for at least 72 h, and the slow release from the micelle revealed a high affinity of AuL12 towards the micelle core. Targeted micelles showed a marked decrease in cell viability when compared to non-targeted micelles or a free drug, indicating a beneficial influence of the targeting moiety. Hiding the drug within the liposomal or micellar structure has the potential to ameliorate the systemic toxicity of the drugs. 

Monomethyl auristatin F (MMAF) is one of the highly efficient anticancer drugs, however, its application is limited due to its significant systemic toxicity. The success of Adcetris, a targeted MMAF–antibody conjugate, stimulated researchers to search for a way to deliver the drug specifically to the target site, thus maximizing the drug accumulation in malignant tissue. This, in turn, can ameliorate systemic toxicity and allow full exploitation of MMAF anticancer potential. Following the abovementioned concept, Kim et al. used polymeric micelles based on block copolymer of poly(ethylene glycol) and poly(1,4-phenylacetone dimethylene thioketal) (PEG-PPADT), tethered with BBN [69]. As expected, MMAF@BBN-PEG-PPADT showed higher anticancer activity than MMAF@mPEG-PPADT and free MMAF. The targeting peptide was shown to play an essential role in the enhancement of cellular uptake in vitro, implying promising anticancer efficiency and improved toxicity in vivo.

An emerging alternative to liposomes and micelles is represented by solid lipid nanoparticles (SLN) and nanostructured lipid carriers (NLC). These constructs are based on the lipid-matrix core, composed of either solid crystalline lipids (SLN) or solid and liquid lipids with less ordered crystalline arrangement (NLC), surrounded by a self-assembled layer of surfactants, stabilizing the whole construct. They offer improved drug encapsulation capacity and even better stability compared to typical micellar and liposomal systems [63,84]. GRPR-targeted SLNs loaded with DOX were reported by Wang and co-workers [63]. Their system demonstrated an excellent in vitro cytotoxicity and in vivo antitumor effect both in MCF-7/MDR breast cancer cell lines and breast cancer animal models—the tumor inhibition rate of BBN-DOX/SLNs in mice was approx. 81%. However, drugs are not the only compounds that can be hidden inside the vesicular cavities. Du and Li have taken this system to the next level—they have complemented their DOX-loaded nanostructured lipid carrier with DNA, for which NLCs are a very effective delivery vehicle [21]. The formulations reached over 67% of transfection efficiency at 72 h, with targeted carriers outperforming the non-targeted counterparts in the lung cancer model both in vitro and in vivo. Such combination therapy may be a solution to multidrug resistance issues due to the synergistic anticancer effect of a drug and the genes delivered.

### 4.2. Gold Nanomaterials

Among various delivery vehicles for therapeutic compounds, gold nanostructures have been one of the most widely studied because of the high degree of biocompatibility, ease of processing, and functionalization, as well as the possibility of size, shape, and surface optimization to suit stringent requirements of targeted therapies [85]. The surface chemistry of gold is relatively well understood and provides the opportunity for stable bonding with ligands ranging from synthetic polymers through proteins (such as antibodies or enzymes) to DNA fragments up to drugs and biosensing molecules. The aspect of interest for many research teams remains, however, the possibility of efficient conjugation with targeting moieties that could enhance cancer treatments’ safety and efficacy through drug internalization mostly into the tumor cells. Moreover, when gold nanostructures are amassed in the tumor and then are treated with electromagnetic radiation from, e.g., a laser, they generate a substantial amount of heat and, as a result, damage the malignant cells without affecting healthy cells surrounding the tumor tissue [85]. This is possible only because of the surface plasmon resonance that these structures show, which can be exploited as an additional therapeutic modality called photothermal therapy. Last but not least, it is also worth highlighting that gold nanostructures are able to boost the absorption of ionizing radiation energy leading to radiosensitization. They promote the creation of secondary electrons that spur the formation of reactive oxygen species [85]. The latter prompts supplementary impairment of the DNA, cell membrane, and mitochondria of the cancer cells and further augments cancer cell eradication. All of these features taken together make AuNPs a particularly interesting material for further exploration. 

Hosta-Rigau and colleagues synthesized and functionalized gold nanoparticles (AuNPs) with an RAF peptide analog (Ac-Cys-Ahx-RAF) serving as the anticancer moiety and a BBN analog (Ac-Cys-Ahx-BBN) with the role of targeting the overexpressed GRPR in HeLa cervical cancer cells [73]. They showed that conjugates of RAF derivative and AuNPs significantly decreased the cell viability, which confirms the retention of the RAF peptide anticancer activity upon adding the Ac-Cys-Ahx spacer and conjugation. Interestingly, a significant reduction in cell viability was found both for AuNP conjugates with the BBN derivative as compared to the control (which is explained by the successful receptor-mediated internalization of the conjugates) and for the AuNP-free counterpart, which is somewhat surprising given the known effects of GRPR activation in cancer cells; authors do not comment on that. However, the cell viability for the mixed system—Ac-Cys-Ahx-Lys-BBN/Ac-Cys-Ahx-Lys-RAF@AuNPs—was comparable to Ac-Cys-Ahx-Lys-RAF@AuNPs, and a notable drop in cell viability was observed (53% and 59%, respectively, in reference to untreated samples) for GRPR-positive cancer cells. Furthermore, virtually no toxicity of the system was shown for poorly GRPR-expressing neuroblastoma SHSY-5Y cells. Authors conclude that conjugation of the peptides may lead to changes in peptides conformation, altering their activity.

Different AuNP-based nanocarriers were synthesized and examined in treating both prostate and colon cancers by Chilug et al. They used engineered bombesin and neurotensin-like peptides and checked how these ligands bind to both types of tumors [28]. One of the targeting moieties was [^68^Ga]Ga-DOTA-PEG(4)-BBN(7–14), which, together with its AuNPs counterpart, was subjected to in vitro and in vivo tests. All the newly synthesized constructs showed promising imaging efficiency for colon and prostate malignancies. This is due to the fact that the conjugation of AuNPs with the modified BBN peptide resulted in roughly a threefold increase of the uptake by the colon tumor (9.82% injected dose (ID)/g vs. 3.31% ID/g) observed in nude mice and almost a 3.5 times higher uptake by prostate cancer cells (3.78% ID/g vs. 13.01% ID/g). Follow-up studies were pointed out to be focused on the application of the constructs in positron emission tomography/computed tomography (PET/CT) imaging.

**Figure 10:**
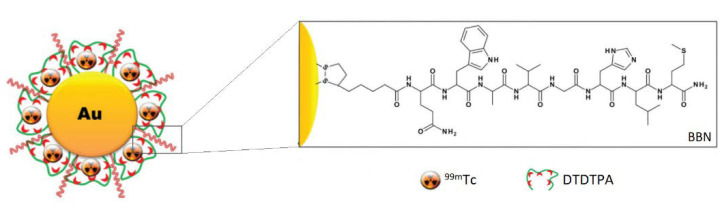
Structure of ^99m^Tc-labeled gold nanoparticles conjugated with BBN and DTDTPA. Adapted with permission from ref. [27]. Copyright 2022, Royal Society of Chemistry.

Another attractive feature that can be introduced with targeted nanotherapeutics is the controlled release of the drugs if those are conjugated to the carrier with suitable cleavable links. Silva et al. postulated that the Au–S and S–S bonds in their BBN–AuNP-based conjugates coated with the thiolated radioisotope-chelating diethylenetriaminepentaacetic acid (DTPA) derivative (2-[bis [2-[carboxymethyl-[2-oxo-2-(2-sulfanylethyl- amino)ethyl]amino]ethyl]amino]acetic acid—DTDTPA) can be selectively cleaved by glutathione (GSH), hence endow the system with the controlled release feature [27]. Interestingly, systems triggered with GSH can be even more selective, since its intracellular concentrations are substantially greater (500–5000 times) than extracellular ones. The γ-counting and neutron activation experiments revealed that BBN-AuNP-DTDTPA radiolabeled with ^99m^Tc released the radioisotope into the GRPR-positive cells in vivo, where a significant amount of GSH is present, while the PC-3 cell culture study with histidine (a powerful chelator for the radiolabeled precursor) did not result in the cleavage of ^99m^Tc from the particles in spite of the BBN presence, indicating its high stability. The in vivo tests also revealed good blood clearance and excretion rate, with most formulations eliminated with urine. Some of the remaining Au and detached DTDTPA-[^99m^Tc]Tc(CO)_3_ did accumulate in the liver and kidneys (10 and 30 % ID/g, respectively, at the 4 h mark) of the CD1 mice. However, this is expected from intravenous administration of the constructs. Therefore, GSH-cleavable BBN-AuNP-DTDTPA (Figure 10) still seems to be a promising platform for GRPR-targeting therapies.

Jimenez-Mancilla et al. addressed an additional, but not less important, aspect of targeted imaging and therapy—drug internalization into the cancer cell nuclei [34]. As a targeting ligand, they used a hybrid peptide consisting of the [Lys3]BBN sequence, short spacer, and additional Tat(49–57) sequence, which promotes cellular penetration and nuclear localization of the construct. The obtained Au-based nanosystem was radiolabeled with two radioisotopes, for which DOTA and HYNIC were used, respectively. ^177^Lu deposits irradiation energy of β particles across hundreds of cancer cells due to low linear energy transfer (LET), while ^99m^Tc targets cellular nuclei adjacent to the carrier due to high LET of Auger electrons [86]. Radiolabeled AuNPs linked with Tat(49–57)-[Lys3]bombesin (^177^Lu/^99m^Tc-AuNP-Tat-BBN) were examined for their biodistribution in PC-3 xenograft-bearing athymic mice upon intratumoral injection. It was found that the Tat(49–57)-[Lys3]bombesin functionalized carrier significantly increased tumor retention of radioactivity compared to its non-targeted counterpart and free radiolabeled targeting peptide. According to the authors, this significant improvement could be attributed to the successful targeting of GRPR, which was also confirmed in receptor blocking in the in vivo experiments. Subsequently, researchers also examined the potential of using the ^177^Lu/^99m^Tc-AuNP-Tat-BBN particles as a thermal ablation system after internalization into the PC-3 cells and found it very effective not only in preventing cell proliferation but also in decreasing cell viability (down to about 1.3%), which, together with the near-infrared (NIR) fluorescence emitted from the conjugates, make them applicable for both targeted radiotherapy and plasmonic photothermal therapy.

AuNPs can also serve as a core in more complex constructs where the metal nanoparticle is capped with various functional moieties. Such hybrid AuNPs, coated with water-soluble chitosan (WSCS) and [Lys1-Lys3(DOTA)]bombesin (DOTA-BBN), were prepared by Tangthong et al. to target GRPR-overexpressing PC-3 and LNCaP prostate cancer cells selectively [81]. Upon 48 h incubation, the examined AuNPs-WSCS-DOTA-BBN particles showed half maximal inhibitory concentration (IC_50_) values of 65.89 and 60.73 μg/mL, respectively, against PC-3 and LNCaP cells (compared to IC_50_ for AuNPs-WSCS being over 100 μg/mL) without any drug loaded; they also remained stable in biological media and proved safe for healthy HAEC aortic endothelial cells. Follow-up studies by the same team explored AuNPs reduced in situ within WSCS anchored with gallic acid (GA) and conjugated with DOTA-BBN [51]. The AuNPs-WCS-GA-DOTA-BBN exhibited an increased internalization into PC-3 cells when compared with the GA-free (previous study) or BBN-free conjugates as well as 2.4-fold lower IC_50_ values (5.94 μg/mL vs. 12.05 μg/mL) after 48 h in comparison to AuNPs-WCS-GA. These findings confirm the targeting effect of the BBN analog incorporated into the system. GA also showed good anticancer properties, and, although more potent when administered alone, considering its negative impact on healthy cells, such a targeted formulation seems very promising for more selective and thus more efficient carcinoma management, especially if the loading concentration was increased above ~15%, which was used in the described study.

Most Au-based biomedical constructs are based on spherical particles, but it is certainly not the only available architecture of gold nanostructures; Heidari and colleagues exploited gold nanorods (GNRs) [48]. They have designed and successfully synthesized GNRs conjugated with a universal binding sequence-derived BBN analog through Nanothinks acid linkers and subsequently coated it with PEG for increased biocompatibility. The study on T47D cells showed that, at the 24 h mark, 12% or 50% of GNR-PEG or GNR-BBN-PEG, respectively, was absorbed into the cells, which is a significant improvement and a potential indication of the receptor-mediated nanoformulation internalization. Additionally, 24 h after NIR laser irradiation of cancer cells incubated with GNR-BBN-PEG, nearly all of them were eradicated, which was not observed for any of the control samples (including cells incubated with GNR-PEG and treated photothermally). Biodistribution analysis in BALB/c mice revealed a doubling of uptake into the tumor tissue and a doubling of the photothermal treatment efficiency for GNR-BBN-PEG intravenously injected mice compared to subjects injected with the BBN-free control. What is important, 15 days after GNR-BBN-PEG and photothermal treatment, 8/10 mice exhibited complete tumor resorption and remained healthy 60 days post-treatment with no carcinoma regrowth, while the entirety of the control group medicated with GNR-PEG was dead by day 45. These results show a substantial improvement in the efficacy and safety of the treatment through the BBN-mediated targeting of GRPRs.

Gold nanorods were also the object of interest in the study carried out by Xu and co-workers [82]. They proposed a novel synergetic strategy combining GRPR-targeted delivery with photothermal effect derived from GNRs and thermodynamic therapy, based on a heat-labile cytotoxic free radical donor (2,2′-azobis[2-(2-imidazoline-2-yl)propane]dihydrochloride—AIPH). Upon heating, produced by NIR laser irradiation of GNRs, AIPH generated oxygen-independent radicals in situ, eradicating the cancer cells while sparing healthy tissues exposed only to intact AIPH. Loading the compound to the gold nanostructures was achieved with the mesoporous silica shell deposited on the surface of the GNRs. Studies on the in vitro prostate cancer model (PC-3 cell line) showed highly specific uptake of BBN-modified, mesoporous, silica-coated gold nanorods containing AIPH (MGN-A-BBN) and significant apoptosis of the cancer cells upon laser irradiation, resulting from heat and release of free radicals. In the mice model of subcutaneous prostate cancer xenografts, MGN-A-BBN with laser treatment almost completely eradicated the tumors after 21 days of treatment, while the mouse body weight and key blood biochemical parameters remained within a biosafety range—it proves the high efficacy and satisfactory biocompatibility of the MGN-A-BBN in vivo.

All of the presented studies provide an interesting insight into the use of AuNPs of different architectures and modifications for cancer treatment and undoubtedly reveal the importance of BBN in directed drug delivery into carcinomas. Some formulations [27,81] would, however, require further examinations to establish whether the targeting moiety provides a sufficient anchor for the carrier to eliminate or at least mitigate the nonspecific binding into organs unaffected by the tumor and, hence, provide a less invasive treatment.

### 4.3. PLGA Nanoparticles

Polymers are widely known for their versatility and impressive variety; therefore, unsurprisingly, they have interested scientists working on drug delivery systems. A broad range of polymers has already been tested and proved to be biocompatible and biodegradable [87,88], and many can be tuned to have stimuli-sensitive properties [89]. Most importantly, they can be combined with anticancer substances and targeting units for effective and safe tumor therapies.

**Figure 11:**
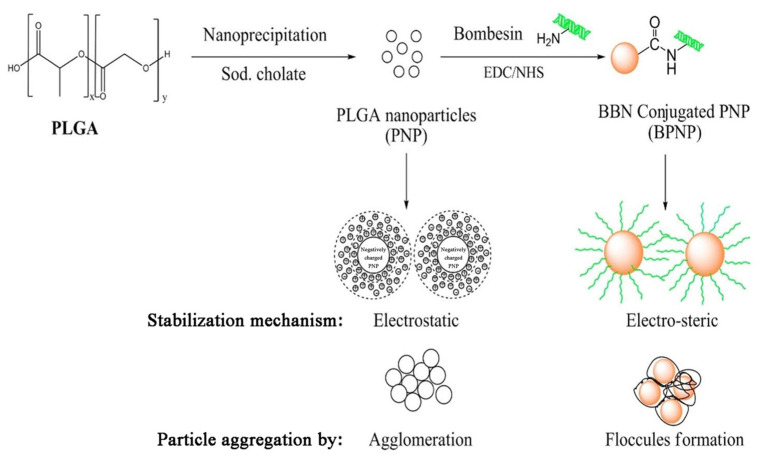
An exemplary synthetic procedure for the preparation of PLGA nanoparticles (nanoprecipitation), surface conjugation of bombesin (BBN) to the nanoparticle surface, as well as the stabilization and aggregation mechanisms for pure and conjugated nanoconstructs. Adapted with permission from ref. [17]. Copyright 2022, Elsevier.

Among biomaterials studied, poly(lactic-co-glycolic) acid nanoparticles seem to be of the greatest interest for the preparation of nanoparticles (NPs) for therapeutics. Kulhari et al. synthesized biodegradable PLGA carriers by a nano-precipitation method and successfully grafted them with BBN through amide bonding (Figure 11) [17]. The PLGA–BBN conjugate exhibited improved stability and aggregation resistance in relation to pure PLGA nanoparticles. Furthermore, the construct durability was examined through BBN release studies and revealed a slow peptide discharge of 6.8% within 1 h (under acceleration conditions), followed by around 12.8% being lost within 24 h, suggesting a satisfactory level of stabilization through covalent bonding. The research continued with experiments on DTX-loaded, BBN-conjugated PLGA particles prepared by the same method, with sodium cholate as the surfactant and BBN conjugated to the surface of already-loaded particles [18]. Formulation allowed for a steady drug release over the period of 120 h, in contrast to pure drug suspension or Taxotere^®^, which showed burst release of the drug within 10 h. Simultaneously, as in the case of most drug-loaded formulations, the initial 24 h was when the greatest amount (~21%) of DTX was released.

Furthermore, a bigger amount (36.2%) of the drug was released into the medium with lower pH (sodium acetate buffer, pH 5) as opposed to PBS with pH 7.4 (31.6%), which appears advantageous for targeted delivery into the tumor, since the pH of cancer environment is known to be acidic [90]. In vitro analysis of the DTX-PLGA-BBN cytotoxicity in GRPR-positive breast cancer cells (MDA-MB-231) indicated a significant improvement in efficacy over the free drug, Taxotere, and DTX-PLGA, with IC_50_ values being, respectively, 35.53 ng/mL for the targeted nanocarriers, 142.24 ng/ml for the non-targeted DTX-loaded NPs, and more than 375 ng/ml for DTX and Taxotere^®^. The above data were very promising for concentrations as low as 3.125 ng/mL, but if the dose is increased, no advantage in toxicity was observed for the BBN-conjugated NPs.

Further studies on DTX-PLGA-BBN nanoparticles were focused specifically on human prostate cancer [80]. Using the same nanoconstructs, scientists investigated DU-145 and PC-3 cells and found IC_50_ values of 241.89, 126.04, and 77.41 ng/mL for DU-145 cells, respectively, for DTX, DTX-PLGA, and DTX-PLGA-BBN. In PC-3 cells, the values given for the samples in the same order were 122.95, 74.44, and 45.02 ng/mL, which indicated a clear and substantial increase in cytotoxicity for the examined nanoformulations. Intravenous administration of Taxotere^®^, DTX-PLGA, or DTX-PLGA-BBN to BALB/c mice (at a dose of 10 mg/kg body weight) revealed slower drug clearance and larger plasma half-life of the NP formulations, but no significant differences between DTX-PLGA or DTX-PLGA-BBN, confirming a similar stability level from the previous studies [17]. Biodistribution analysis indicated a rapid and rather high absorption of the nanoconstructs into the reticuloendothelial organs, with DTX-PLGA and DTX-PLGA-BBN accumulated mostly in the spleen and lungs, as compared to high drug concentrations observed in the kidneys for Taxotere^®^. Although, at the 12 h mark, the DTX concentrations per gram of tissue were mostly below 1%, these results certainly do not seem promising in terms of targeted delivery, and the only advantage indicated by the authors seems to be the DTX absence in the brain during the course of the study.

The use of other polymers, although widely studied for drug delivery, has not been examined to a great degree for GRPR targeting. This might be because liposomes and micelles based on polymers offer better properties for loading, conjugation, or drug release and can be used for numerous therapeutics regardless of their hydrophobicity/hydrophilicity. All the above-mentioned studies present some potential for improving tumor-specific drug delivery, but, unfortunately, none provided sufficiently satisfactory results. In Section 5.3, we showcase other interesting polymeric formulations with theranostic applications. The carrier’s architecture was designed to allow particle tracking using various diagnostic modalities and improve drug delivery into cancer cells.

### 4.4. Other Materials and Structures

In light of the need for new therapeutic strategies, an out-of-the-box search yields sophisticated formulations brought together by a cross-domain approach. Zhang et al. have developed genetically engineered elastin-like peptides (ELP), with GRPR-binding motif included in the N-termini of the peptides (ELP-GRP), expressed by the *Escherichia coli* strain transfected with plasmid vectors prepared with molecular biology techniques [67]. Their peptides were able to form monodisperse temperature-responsive micellar structures with sizes around 40–55 nm, capable of encapsulating a hydrophobic fluorescent label, 1-anilinonaphthalene-8-sulfonic acid (1,8-ANS). Confocal microscopy revealed that these structures were efficiently internalized in vitro in GRPR-positive PC-3 and DU-145 cells, but not in GRPR-negative 293T cells; high uptake in prostate cancer cells was mediated by receptor–ligand interaction, as proven by increased Ca^2+^ release upon incubation of cells with receptor-activating ELP-GRP micelles.

**Figure 12:**
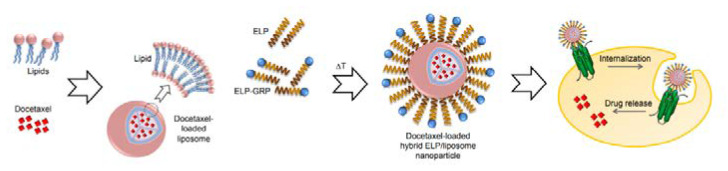
Scheme of the process of specific targeting of prostate cancer cells with hybrid elastin-like polypeptide (ELP)/liposome nanoparticles. Adapted from [79].

Subsequently, Zhang and colleagues took their ELP nanostructures to the next level and constructed innovative hybrid ELP/liposomal nanocarriers, combining the liposomal formulation’s advantages with the ELP-GRP targeting potential to form a docetaxel delivery vehicle with high specific binding and improved cytotoxicity [79]. As a result, 40–200 nm monodisperse nanoparticles with neutral zeta potential showed high entrapment of DTX and GRP motif on the surface and enhanced specific binding of their hybrid carriers to prostate cancer cells, but not to GRPR-free cells (Figure 12). Moreover, targeting improved the cytotoxicity of the DTX-loaded nanostructures.

Interestingly, the authors have also elaborated on an important factor frequently overlooked, namely the influence of surface charges described by the zeta potential on the fate of the nanoparticles in vivo. In general, the surface charge is desired due to the fact that it stabilizes the nanoparticles in the solution. However, one should keep in mind that particles with negative potential (below −10 mV) would trigger the plasma proteins’ binding and opsonization, leading to an uptake in the reticuloendothelial system (RES) and fast clearance from the bloodstream. On the other side of the spectrum, particles with high surface potential (more than 10 mV) were found to aggregate in serum. These are the neutral particles (with a surface potential between −10 mV and 10 mV) that possess the most desired in vivo characteristics (least RES interactions, minimized nonspecific uptake, and the longest time of blood circulation), despite the fact that this is not the most desirable characteristic from the colloidal stability point of view.

**Figure 13:**
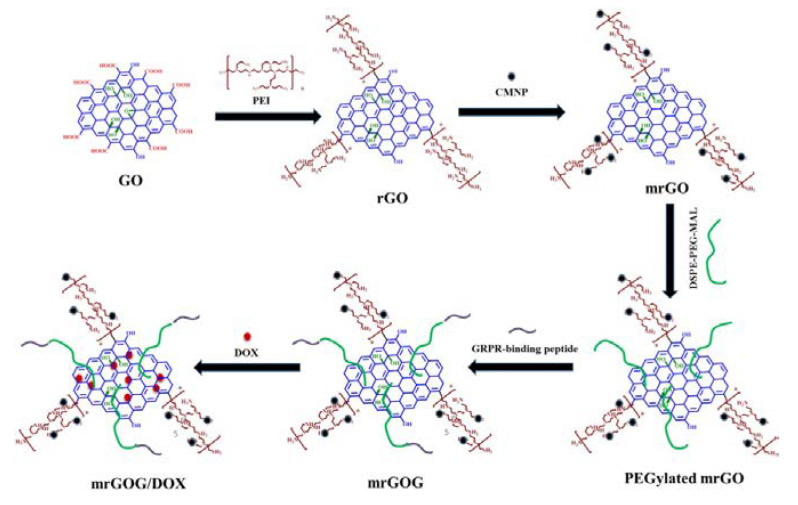
The process of preparing BBN-conjugated, DOX-loaded magnetic reduced graphene oxide NPs (mrGOG/DOX). Reprinted with permission from ref. [62]. Copyright 2022, Elsevier.

Synergistic strategies for cancer treatment can also be an important contribution to current therapeutic needs. In research published by Dash and co-workers, a multifunctional system based on the combined action of a magnet, laser, and drug in magnetic reduced graphene oxide was demonstrated [62]. The magnetic nanocomposite (mrGOG) was obtained by conjugation of reduced graphene oxide with magnetic iron oxide nanoparticles, PEG moieties, and bombesin-like GRPR-binding peptide (Figure 13). Following functionalization, the construct was also loaded with DOX using π–π stacking between the drug and graphene rings (mrGOG/DOX). Dual targeting of the construct based on GRPR-binding peptide supported by magnetic targeting led to the successful delivery of DOX to cells, where the drug was successfully released in the endocytic compartments with acidic pH. In combination with NIR laser irradiation, nanoformulation significantly reduced the cell viability of U87 glioblastoma cells in vitro. In vivo studies confirmed these results: mrGOG, combined with magnetic targeting and laser irradiation, significantly slowed cell proliferation and tumor growth in mice bearing tumors with U87 xenograft.

## 5. Molecular Imaging

Table 2 provides examples of the contemporary (ca. 2006 to date) advances in the research concerning molecular imaging applications of nanocarriers functionalized with GRPR-targeting moieties.

### 5.1. Gold Nanomaterials

**Figure 14:**
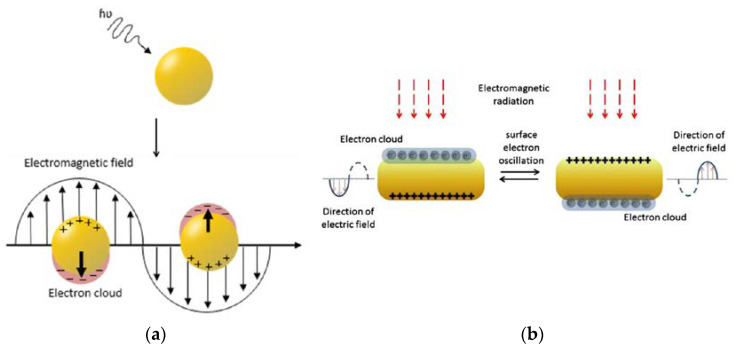
Schematic representation of the surface plasmon resonance (SPR) effect in (**a**) gold nanoparticles (adapted from ref. [99]) (**b**) gold nanorods (adapted with permission from ref. [85]. Copyright 2022, Elsevier).

Gold nanomaterials are of huge importance when it comes to imaging. They have excellent optical properties of light absorbance and light scattering; not only do they work in a visible-light spectrum, but they are also able to operate in the near-infrared range [85]. Furthermore, depending on the extent to which the gold nanoparticles are modified, the electron charge density on the surface of an individual nanoparticle can differ, and this phenomenon is related to the already-mentioned feature of AuNPs—surface plasmon resonance (SPR) [95]. This phenomenon leads to the creation of an electromagnetic field at the surface of the gold nanoparticle (Figure 14) and to surface-enhanced optical properties. Because of the high atomic number of gold, Au nanostructures can also find their application as imaging contrast agents. In fact, numerous studies [96,97] demonstrated that these materials can work as contrast agents in photoacoustic imaging [98], or computed tomography (CT), to name but a few. However, upon further functionalization, they may serve as excellent biocompatible carriers in multifunctional imaging nanosystems.

Photoacoustic imaging (PAI) relies on the formation of a sound wave following the absorption of light, hence offering high ultrasonic resolution and strong optical absorption. It was shown previously that gold nanorods, due to very strong light absorption compared to organic dyes, provide potent photoacoustic contrast and boost the diagnostic potential of PAI in vivo [98]. For this reason, Heidari and co-workers investigated bombesin-derivative-functionalized GNRs as potential nanotracers in PAI [43]. Their research proved that their nanomaterial was stable in human blood serum and able to selectively bind with GRPR-positive cells both in vitro in the T47D breast cancer cell line and in vivo in breast cancer tumor xenografts in BALB/c mice. Moreover, they showed that the uptake augmented in time, reaching the maximum signal intensity 8 h after intravenous injection.

Chanda and co-workers also focused on GNRs and their great photophysical properties, but extended their research to targeting both breast and prostate tumors [93]. Their goal was to design and develop bombesin-conjugated nanoparticles in order to overcome contemporary issues with breast/prostate tumor imaging. To do so, they elaborated on the mechanism of the internalization of targeted nanostructures. The nanorods were of 40 × 13 nm size and functionalized with various BBN derivative ratios. At the highest level of decoration, the nanomaterial was shown to be chemically stable in various media. Targeted GNRs were effectively taken up by GRPR-positive cells (T47D and PC-3) by means of receptor-mediated endocytosis, but not phagocytosis. This has been proven by performing dark field light-scattering, fluorescence imaging, and transmission electron microscopy (TEM) measurements. When the fluorescence and dark-field scattering images overlapped, it could be clearly seen that the GNR-BBN carriers are located in the cytosol. This fashion was not observed for GRPR-negative NIH-3T3 cells, where significantly fewer GNRs were internalized, phagocytosis played a major role, and nanostructures were trapped in endosomes.

Later, the same group of researchers investigated the performance of gold nanoparticles modified with different levels of bombesin substitution [72]. Their starch-encapsulated AuNPs were found to be the most stable, with the greatest amount of bombesin attached to the surface. Additionally, when the PC-3 cell binding affinity was tested, it turned out that when more bombesin molecules were attached to AuNPs, a 3.3-fold decrease in the IC_50_ value (2.45 μg/mL vs. 8.10 μg/mL) was observed. In order to assess the in vivo performance, radioactive ^198^Au was utilized, and the severe combined immunodeficient (SCID) mice were injected with bombesin-altered nanoplatforms. Typical biodistribution was shown, with around 0.5% ID/g accumulation in prostate cancer tumors and promising diminished amassment in the liver despite a slightly bigger particle size than those reported in the earlier literature reports. Nanoparticles were also shown to significantly increase contrast in tumor imaging in CT after intraperitoneal administration of the nanocarrier. The signal was multiple times more intense than required to serve as a prostate cancer CT agent, and it could be seen to deteriorate only 6 h after injection. Surprisingly enough, a very weak signal could still be detected after 48 h, suggesting some enhanced retention in the tumor.

The great potential of gold nanoparticles as a contrast agent in X-ray imaging was investigated by Salouti and Saghatchi [44]. They found that their PEG-coated AuNPs functionalized with pan-bombesin had very good optical stability when introduced into blood serum, and, by performing appropriate binding studies, they have evidenced that their conjugate could selectively target breast cancer tumor tissue (T47D cell line). Furthermore, researchers found out that this formulation could be successfully used in radiology, as the breast tumor in the BALB/c mice could be easily distinguished after 6 h, and the clear signal was retained up to 8 h post-injection. Afterward, no obvious accumulation trend was seen, and thus, it suggests that the construct clears from the tumor quite quickly—the signal in the X-ray images becomes somewhat faded. Furthermore, no amassment was observed when it comes to non-targeted NPs, proving the binding specificity of nanovectors.

Pretze and colleagues have exploited ultra-small (3 ± 2 nm) GRPR-targeted AuNPs as biocompatible carriers for fluorescent probes in confocal fluorescence microscopy [91]. Their particles were functionalized with bombesin and the NIR dye SIDAG. The performance of the particles modified with bombesin showed a good uptake by PC-3 cells when compared to the free targeting ligand. The results of in vitro experiments on prostate cancer cells revealed that the construct provided a good fluorescent signal, and the particles could be nicely visualized after 24 h of incubation. Subsequent in vivo studies on healthy mice 21 h post-injection showed that the carrier accumulated mainly in the liver and spleen, however, when a threefold higher dose was tested on mice with PC-3 prostate cancer xenografts, it was found that SIDAG-BBN-AuNPs amassed in the tumor mass between 6 and 24 h and allowed tumor visualization. Moreover, a preliminary in vitro study also proved utility of the system as an efficient contrast agent for CT with good signal-to-noise-ratio, even when the concentration of AuNPs was reduced by a factor of 13 compared to fluorescent imaging.

Following the success of the fluorescent system, Pretze et al. further developed the above-mentioned nanosystem to visualize prostate cancer via PET scanning [92]. They again used very small AuNPs and conjugated them with an NIR fluorophore and targeting ligands, although, this time, greater emphasis was placed on the functionalization with the chelator (NODAGA). Radiolabeling with ^64^Cu was achieved with high radiochemical yield and great stability in vitro. The authors provided an interesting explanation regarding the successful targeted delivery of the construct: since elemental copper binds to the DNA, it causes more cell damage than the nanocarrier-bound Cu, which, in turn, is retained in cell plasma and does not reach the nucleus. Cell viability experiments have shown higher cell toxicity of free Cu compared to Cu-labelled AuNPs, hence the conclusion about the successful targeting of the construct. To confirm in vivo whether the cancer-detecting units allowed visualization of the tumors, nanoplatform was administered intravenously into SCID mice. Distinguishable signals were obtained even 3 h after injection of the moieties for PET/CT imaging, and the prostate tumor could be easily recognized. However, fluorescence imaging did not yield the expected results, as the signal was too intense at first, and only after 24 h after injection could other organs be discerned. The biodistribution studies also confirmed a typical AuNPs demeanor where the moieties accumulated mostly in the liver and spleen.

Imaging of prostate tumors was also the goal of Mendoza-Sánchez and colleagues, who wanted to exploit single-photon emission computed tomography (SPECT-CT) [23]. To do so, they synthesized a multifunctional construct based on AuNPs functionalized with HYNIC for ^99m^Tc chelating. Selective binding to PC-3 prostate cancer cells was achieved by H_3_N^+^-Lys3-bombesin tethered to the surface gold atoms. The obtained nanovector, of ca. 20 nm size, was proven to be highly stable in human serum, and its radiochemical purity exceeded 95%. It was shown that targeted NPs could accumulate in the tumor 1.7 times better than unfunctionalized ones (36.93% vs. 21.58% of total injected activity) in vitro. This trend was confirmed in vivo by microSPECT/CT imaging on athymic nude mice prostate with tumor xenografts. Malignant growth could easily be observed, as the contrast between the carcinoma and the surrounding tissues was considerably enhanced 1 h post-injection (6.39 ± 0.83% injected activity). Engrossingly, the uptake by the tumor could be noticed as early as 30 min after the material administration. When the test was performed again, 24 h after injection—no signal was found in microSPECT/CT—thus, full clearance was demonstrated in less than a day. Nevertheless, not differently than in many other GRPR-targeting projects, the typical accumulation region during this study turned out to be the pancreas (39.83 ± 2.76% injected activity at 1 h) as there are approximately seven times more GRPR sites to bind than in prostate tumor.

Still, further elaboration on this construct was shown by Ocampo-García and colleagues—by altering and, therefore, boosting the current design, they developed the stable kit for ^99m^Tc labeling of a multifunctional receptor-specific system based on gold nanoparticles [22]. The kit comprised two vials: one with a lyophilized chelating moiety (HYNIC derivative) and the second with a 20 nm AuNPs-biomolecule solution; studies demonstrated the 6-month stability of the abovementioned kit. Moreover, the bombesin-functionalized nanosystem obtained from the kit ([^99m^Tc]Tc-AuNP–Lys^3^-bombesin) showed 1.8 times higher uptake in prostate cancer cells than non-targeted NPs. This fashion was confirmed by conducting in vivo studies using micro-SPECT/CT imaging; the prostate cancer considerably took up the AuNP-based nanosystem. Therefore, the newly synthesized kit was claimed to have well-fitting properties in order to be used as a target-specific agent for molecular imaging of tumors expressing GRPR.

### 5.2. Iron Oxide Nanoparticles

Iron oxide nanoparticles (IONPs) are one of the two most important inorganic nanomaterials, next to gold nanoparticles, owing to their outstanding magnetic properties [100]. GRPR-targeted cancer imaging efficiency of iron oxide nanoparticles is being investigated to a slightly lower extent than gold nanoparticles, but, considering their clinical success [101], they still pose a great importance in contemporary cancer management, especially in bioimaging of malignant tumors. The most commonly used IONPs are SPIONs—superparamagnetic iron oxide nanoparticles. Their most recognizable property is reversible magnetization [102], making them a promising material. When the external magnetic field is applied, SPIONs can be easily guided to the tumor location, and when it is removed, no magnetization is seen. Moreover, SPIONs may supply the negative MRI contrast in T_2_-weighted magnetic resonance imaging (MRI) [103]. This is particularly important in the intracellular magnetic labeling of tumor cells. When the iron-based nanoparticle is conjugated with the targeting ligand, the specific binding of the probe to the malignant tissue can be visualized in a more efficient manner.

There were many reports on research conducted on the performance of iron oxide nanoparticles in bioimaging. However, the yield of successful operations of them was mostly examined in prostate, breast, and pancreas cancers. Jafari created bombesin-modified SPIONs coated with carboxymethyl dextran for breast cancer magnetic resonance imaging [45]. Their construct was stable in human blood serum for up to 24 h of incubation and not toxic to breast cancer cells (T47D) up to 30 µg/mL. They have demonstrated that their carrier could selectively bind to the GRPR-expressing cells but not to cells lacking the receptor. Furthermore, in vivo MRI experiments in tumor-bearing BALB/c mice revealed favorable properties of their construct regarding the enhancement of the contrast of the tumor imaging with constantly excelling the MRI signal until 13 h after nanocarrier administration and excreting the manufactured contrast agent after 30 h. After that, the T_2_ relaxation time returned to its preinjection level. As a result, the novelty can be used as an attractive material for the T_2_ MRI contrast agent.

Negative contrast in T_2_-weighted MR imaging inspired Montet et al. to develop a highly out-of-the-box idea for imaging pancreatic ductal adenocarcinoma [70]. Their inversed approach was quite unique: they used superparamagnetic cross-linked iron oxide (CLIO) nanoparticles functionalized with bombesin to target healthy pancreatic tissue rich in GRPR (in contrast to malignant tissue). Their nanosystem selectively decreased the T_2_ parameter in the normal pancreas, thus giving rise to the enhancement of the signal shown by the pancreatic tumor when compared to the pre-contrast image. Fluorescent microscopy of cryosections of the organs of interest, performed following MRI, confirmed these finding, therefore proving the material to possess the desired properties for medical tumor imaging.

**Figure 15:**
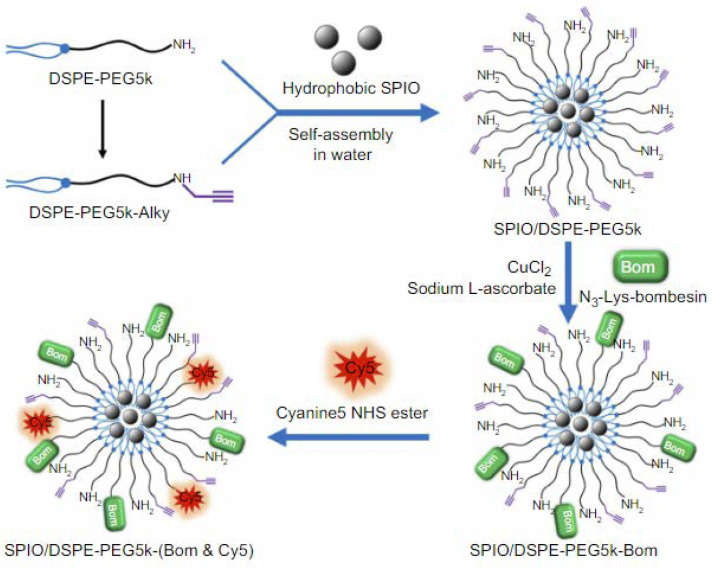
The steps of the synthesis of PEGylated, superparamagnetic iron oxide (SPIO) nanoparticles coupled with Lys3-BBN and Cy5 fluorophore. Adapted from ref. [60].

The performance of iron oxides was also tested by Martin and her colleagues in the management of prostate cancer [46]. They have prepared dextran-coated SPIONs, functionalized with fluorescent rhodamine derivative as well as clickable azides—they have demonstrated click cycloaddition as a means of the successful functionalization of the nanocarrier with BBN peptide. Furthermore, cell-binding studies proved that the PC-3 cells can take up newly constructed carriers significantly more efficiently than unfunctionalized SPIONs. As for the fluorescence investigations, only after 2 h of incubation of the prostate cancer cell line it could be observed that an intense signal could be detected from PC-3 cytoplasm, reaching over 300% as intense a signal as the one obtained from non-targeted NPs. Thus, their nanotransporter can serve as an excellent magnetofluorescent bioimaging probe for prostate carcinoma. Li et al. in turn, exploited SPIONs coated with DSPE-PEG, which provided the means for further functionalization with NIR fluorophore (Cy5) and bombesin analog [60]. The obtained nanomicellar system (Figure 15) was tested for its dual-modality imaging properties in breast cancer diagnosis. Not surprisingly, a strong signal was detected by fluorescence microscopy after the MDA-MB-231 cells were incubated with the nanomaterial in vitro, indicating that the newly synthesized material demonstrated high breast cancer targeting capabilities.

Furthermore, when this GRPR-targeted nanocarrier was injected into the BALB/c nude mice with MDA-MB-231 breast cancer xenografts, high-quality magnetic resonance and NIR fluorescence (NIRF) images of tumors were obtained with excellent contrast. These results prove that the construct is a suitable multimodal imaging probe, since the non-targeted samples did not yield any significant improvements in the signal received from either MRI or NIRF imaging.

An unusual method was undertaken by Lee and co-workers, where they combined iron oxide nanoparticles with nanostructured glycol chitosan (GC) to create a hybrid nanomaterial (IO-BC-NAHis-GC) for applications in magnetofluorescent imaging of prostate cancer tissue [71]. The polymeric nanostructure of a size range between 35 and 97 nm was obtained by self-assembly and further coupled with a Cy5.5 fluorescent dye. The loading of IONPs was achieved due to hydrophobic interactions between the oleic acid shell of IONPs and the hydrophobic core of the nanostructured GC, and the size range of the final product was 45–90 nm. In vitro studies evinced that those hybrid NPs are prone to bind to GRPR-positive cells. Although MRI was not part of this study, all the conducted imaging studies strongly support the statement that this hybrid material holds great potential in the field of prostate cancer fluorescent and magnetic resonance imaging.

Multimodal imaging properties of SPIONs can also be exploited by adding a radioactive component to the construct. Following this concept, Hajiramezanali and co-workers synthesized chitosan-derivative-coated SPIONs, conjugated with bombesin and isotope-chelating DOTA moiety [54]. Radiolabeling of iron oxide nanoparticles with ^68^Ga rendered the formulation suitable for both MR and PET imaging. The examined product ([^68^Ga]Ga-DOTA–BN–TMC–MNPs) turned out to have very good activity obtained from gallium radiolabeling with decent biological stability both in vitro and in vivo. Furthermore, it was found that DOTA–BN–TMC–MNPs could be used successfully as a T_2_ MRI contrast agent for detecting breast cancer in vivo, since the concentration of iron at the tumor site was high enough to be discerned. Additionally, PET studies indicated excellent visibility of breast cancer xenografts due to the significant uptake of NPs at that site.

Lahooti and colleagues chose an analogical approach, which proposed ultra-small SPIONs capped with 3-triethoxysilylpropyl succinic anhydride (TEPSA) [36]. In their research, they conjugated the NODAGA chelator and bombesin peptide to PEGylated USPIONs to selectively target breast cancer cells (MDA-MB-231 and MCF-7) and deliver ^68^Ga-derived activity. The sizes of the obtained nanoparticles did not exceed 24 nm, depending on the compounds coupled with NPs (transmission electron microscopy ~7 nm; dynamic light scattering ~22 nm). This material showed a great affinity for GRPR-positive cells in vitro and increased tumor uptake in vivo upon intravenous injection, hence leading to improved tumor contrast in MRI images. In fact, the conjugation of the ligands to USPIONs affected the relaxivity—over time, after the injection of the nanoformulation, the T_2_ parameter value in the breast tumor diminished by around 25%. Furthermore, PET/CT imaging 2 h after injection showed tumor uptake, however, it was observed that, most likely due to the very small size or the protein corona formed over the nanoparticle, it was quickly cleared out by the urinary system.

### 5.3. Quantum Dots

Quantum dots (QDs) undoubtedly are instrumental in the imaging of various tumors in contemporary medicine. Relatively high interest in the use of QDs in this area is provoked by the fact that their properties, such as long-lasting photostability, high signal brightness, or the ability of parallel excitation at several wavelengths, are highly favorable in visualizing tumors. So far, in GRPR-positive malignancies targeting, the biggest amount of research is focused on prostate cancer imaging.

**Figure 16:**
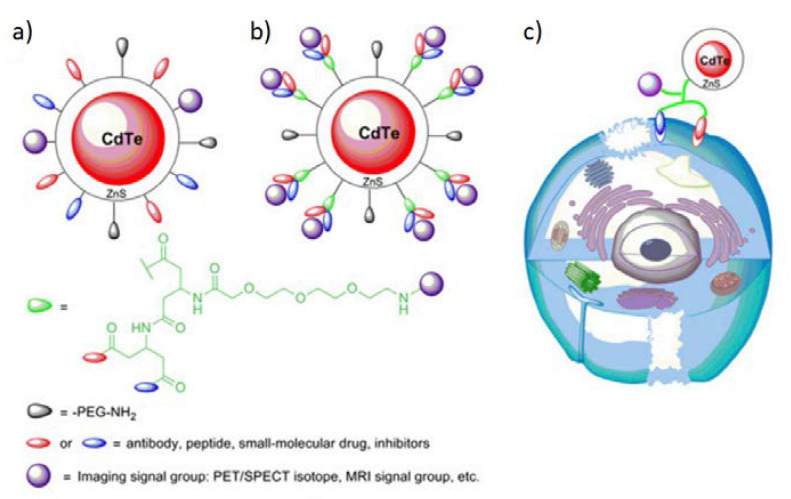
Representation of QD functionalized for in vivo targeting and imaging, including (**a**) the structure of a multiplex modifying multifunctional QD probe, (**b**) the structure of a single modifying multifunctional QD probe, and (**c**) the general idea of enhancing synergistic binding of the heterodimeric multifunctional QD probe. Adapted from ref. [32].

One of the compounds suitable for QDs synthesis is CdTe. Hu et al. employed this material to develop ZnS-coated, bombesin-modified QDs (Figure 16), which could be used to visualize the prostate tumor via NIRF imaging [32]. Additionally, they have equipped obtained nanostructures with PET imaging-suitable signal groups containing ^18^F. In vivo and ex vivo, NIRF studies revealed that the accumulation at the target site proceeds relatively slowly, and the high tumor-to-background ratio (Tu/Mu) was obtained only after 5 h post-intravenous injection, however, the Tu/Mu parameter was still much greater than for the non-targeted quantum dots. When it comes to the assignation of the boundaries between the malignant and healthy tissue by PET imaging, the method turned out to be successful and required only an hour to show the greatest level of radioactivity in the prostate tumor xenograft.

On the contrary, Cai and co-workers investigated orthotopic prostate cancer [20]. They used it to examine the imaging potential of bombesin-functionalized, ^64^Cu-radiolabeled CuS QDs. By virtue of the smart choice of the particle’s composition, authors obtained simple, chelator-free positron-emitting probes suitable for PET/CT imaging. GRPR-targeting bombesin peptide allowed the selective accumulation of the probe in PC-3-KD1 cancer cells in vitro, as shown in the receptor saturation study. However, the greatest value of this research is focused on the above-mentioned model of prostate cancer for examining nanoparticles’ in vivo performances—subcutaneous xenografts fail in accurately mimicking the tumor microenvironment and vasculature; therefore, orthotopic models provide more relevant experimental conditions. The team synthesized BBN-PEG-[^64^Cu]CuS NPs that have shown high accumulation at the tumor site even 1 h after injection in the orthotopic prostate cancer model of Nu/Nu mice and allowed high tumor-to-healthy tissue contrast, resulting in the acquisition of exceptionally clear images.

Another interesting take on QDs was shown by Young and Rozengurt—they checked the performance of quantum dots functionalized with targeting ligands using a biotin-streptavidin system [74]. They tethered biotinylated bombesin to the commercially available streptavidin-modified QDs, emitting fluorescence at 655 nm, and examined them using fluorescence microscopy on bombesin receptor-positive Swiss 3T3 cells, bombesin receptor-negative Rat-1 cells, as well as Rat-1 cells stably transfected with bombesin receptor. The investigations revealed that the conjugation of bombesin peptide to the Qdot 655-streptavidin promotes selective binding of the construct and enables high-quality imaging. Noteworthy, researchers also showed, using another short oligopeptide ligand (biotinylated angiotensin II—ANGII), that conjugation does not influence the intensity of the QD’s signal, and this nanoprobe is superior to low molecular weight fluorescent dye (Cy3) in terms of photostability, proving QDs to be an efficient tool in molecular imaging.

### 5.4. Other Materials

**Figure 17:**
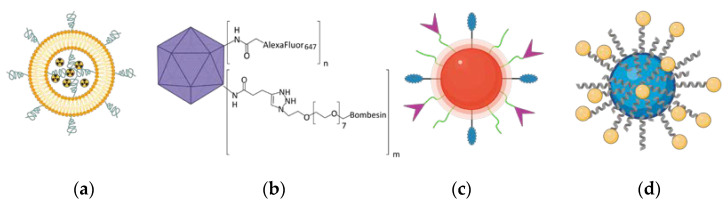
Other representative nanoformulations used for tumor imaging include (**a**) long-circulating, pH-sensitive, BBN-labeled liposomes with ^99m^Tc radiotracer (inspired by [75]), (**b**) dye-labeled, PEGylated cowpea mosaic virus (CPMV) conjugated with bombesin (inspired by [47]), (**c**) PEGylated, Gd_2_O_3_-based functional nanoparticles coupled with bombesin and 5(6)-carboxyfluorescein (inspired by [94]) and (**d**) lanthanide-oleate-based upconversion nanoparticles (UCNPs) functionalized with poly(acrylic acid) (PAA) and coupled with a BBN analog (inspired by [49]). Created with BioRender.com.

Besides the before-mentioned groups of particles, scientists have explored a variety of other nanosystems to target GRPR in bioimaging applications. Liposomes, graphene derivatives, and biobased structures are among the nanomaterials that are gaining more and more attention from the research community (Figure 17). What is important is that some of those are dedicated not only to managing breast and prostate malignancies, but also, for example, head and neck cancer. It clearly shows how broad the spectrum of material science applications in nanomedicine can be.

To begin with, De Barros and colleagues prepared and characterized a liposome containing [^99m^Tc]Tc-BBN(7–14) peptide so as to identify its ability to identify the GRPR-positive Ehrlich tumor [75]. In contrast to most of the research published in the field of cell membrane receptor targeting, in their formulation, BBN ligand was encapsulated inside the liposome in order to protect it from the circulating endopeptidases. Results proved that, despite the unusual design, the carrier can be used successfully in diagnostic applications, as shown by the high uptake of radioactivity by the tumor. Scintigraphy images showed a preferential tumor-to-muscle ratio, supporting the tropism of the liposomes towards the malignancy in the Swiss mice Ehrlich tumor model. The same research team has also published their results on the performance of the aforementioned liposome in targeting breast adenocarcinoma [77]. Their nanocarrier showed sufficient stability in plasma and relatively long circulation in blood. Not surprisingly, although liposomes displayed renal clearance and reticuloendothelial system uptake, there was a moderate uptake after 1 h in the tumor with no reduction after 4 h, which indicated a successful delivery of radioactivity-carrying peptide to the tumor site with the liposomal carrier. On the other hand, free nonprotected peptide showed a twofold lower uptake compared to the liposomal formulation, and the ratio of the tumor-to-other tissue was significantly reduced as well. Similarly to the previous report, scintigraphy images and biodistribution studies evidenced satisfying discrimination between healthy and malignant tissue in the bioimaging of breast cancer.

Another very innovative idea was described by Steinmetz et al., who utilized a naturally-occurring biobased nanoparticle type, a viral nanoparticle based on cowpea mosaic virus (CPMV), to target and visualize prostate cancer [47]. Their construct maintained its structure and integrity upon successful conjugation with an NIR fluorophore (Alexa Fluor 647) and targeting ligand (pan-bombesin analog). The obtained virus-based vehicle (ca. 30 nm) was able to specifically bind to and penetrate the PC-3 cells, contrary to its non-targeted counterpart, both in vitro and in vivo. CPMV nanocarrier could amass in prostate cancer of a 5 mm size, which implies that its use can be efficient in detecting really small malignancies within the body. Furthermore, a clear fluorescence signal coming from the cancer tissue could be seen only after 2 h post-injection, and the tissue surrounding the tumor displayed a negligible fluorescence intensity, proving that the use of targeted CPMV has great potential. Fluorescent imaging was also within the interests of Li et al., however, they used nano-graphene oxide (NGO) as a carrier for further functionalization—the newly synthesized construct was equipped with a Sta-BBN antagonistic ligand and fluorescent Alexa Fluor 750 (NGO-BBN-AF750) [40]. The thorough studies revealed a high binding affinity and specificity towards the oral squamous cell carcinoma (OSCC) HSC-3 cell line and not to the GRPR-negative control, human oral keratinocytes HOK cell line. Having performed immunofluorescence studies, the authors obtained high-quality images and, thus, concluded that NGO-BBN-AF750 is suitable for imaging this kind of tumor.

Although iron oxide is the most popular material for MRI contrast nanoparticles, Cui et al. used a different compound—gadolinium oxide [94]. The primary difference between those two materials is the type of relaxation—while iron provides good contrast for T_2_-weighed visualization, gadolinium is useful in T_1_-weighed image acquisition. Nanoparticles obtained by Cui et al. were shown to function as an immensely efficient T_1_-weighed MRI contrast agent with magnetic properties comparable to the ones of the commercially available gadolinium-based equivalent (Magnevist). Upon functionalization with BBN and carboxyfluorescein (FAM), nanoprobes provided strong green fluorescence when incubated with PC-3 prostate cancer cells in vitro, while this was not the case for the nanoparticles conjugated only with the FAM. This proved the specificity of binding to the PC-3 cell line in vitro. The desirable behavior of gadolinium oxide-based NPs was also corroborated by in vivo MRI and fluorescent imaging studies. While the strongest signal in magnetic resonance was to be seen 2 h post-injection for bombesin-altered specimens, the non-targeted nanoparticles did not demonstrate any considerable result. This was also verified by fluorescent imaging, where the construct showed a very intensive signal and, thus, confirmed the tendency described above.

**Figure 18:**
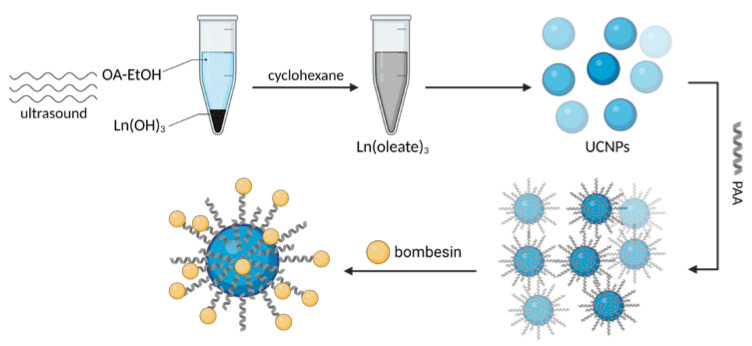
The synthesis of PAA-coated, BBN-labelled upconversion nanoparticles (UCNPs) using lanthanide oleate as the precursor (inspired by [49]). Created with BioRender.com.

Tang and colleagues proposed another multimodal imaging probe to detect and visualize prostate cancer—they used lanthanide oleate complexes as precursors and formed upconversion nanoparticles (UCNPs) based on yttrium, ytterbium, gadolinium, and thulium, capable of absorbing two or more low energy photons and converting them into one emitted photon with higher energy [49]. They modified them with BBN analog (Figure 18) and performed a very broad spectrum of tests, including in vitro and in vivo imaging to examine the targeting abilities of the UCNP-BBN probe. In the in vitro experiments, they used a PC-3 cell line and a GRPR-negative control group. Their findings confirmed the anticipated behavior of UCNP-BBNs, which are selectively bound to prostate cancer cells.

Moreover, upconversion luminescence (UCL), MRI, and CT examinations disclosed that the implementation of the newly synthesized construct greatly enhanced the tumor in vivo visualization in the Nude mice animal model. Interestingly, UCL turned out to be of great importance, as the signal could have been observed only 3 min post-injection and was still detectable after 4 h. For magnetic resonance and computed tomography, the signal amelioration was discerned after 4 h of the administration of UCNP-BBNs, the former method being slightly more efficient, as there was a 25% enhancement of MR signal (for CT: 20% signal intensity improvement).

## 6. Multifunctional Particles for Theranostic Interventions

As already mentioned, theranostic interventions rely on the combination of therapeutic and diagnostic utilities in one nanosystem, as shown in Table 3, based on the contemporary (ca. 2006 to date) advances in this field.

Currently, the easiest (but not the only) way to achieve such an effect is to employ radiation emitted by radioactive nuclides. Radionanomedicine [86] has become a brand-new field of medical science and is continuously gaining more attention, despite sophisticated requirements associated with appropriate facilities assuring radiation safety. Radionanomedicine takes advantage of already established fundamentals of nuclear medicine and simultaneously exploits the achievements of nanomedicine to deliver maximized performance for contemporary cancer management demands. In theranostic formulations, radioisotopes can play different roles, but, practically, in GRPR targeting research, one will find mostly two approaches. γ-emitting nuclides, such as ^111^In or ^67^Ga, provide means for nanocarrier tracking by SPECT-CT. On the other hand, there are isotopes such as ^188^Re or, commonly used, ^177^Lu, which can emit relevant doses of both β^-^ (can cause significant radiobiological effects and lead to cell death) and γ radiation, which makes it the perfect radionuclide for theragnosis.

Usually, the incorporation of radionuclides requires the application of proper complexing moiety. One of the most frequently used chelators is DOTA and its derivatives. Silva et al. designed and synthesized AuNPs tethered with thiolated DOTA (TDOTA) to coordinate Gd^3+^ and ^67^Ga^3+^, potentially useful for MRI and SPECT, respectively [26]. For prostate cancer cell targeting, thioctic acid-terminated bombesin (TA-BBN) was ligated to the AuNP’s surface. Experiments with the PC-3 cell line proved a significant influence of targeting ligands on the construct internalization in vitro during up to 3 h of incubation. Moreover, upon exposition to 2 Gy of external gamma irradiation, a clear radiosensitization effect was found, as PC-3 cells incubated with targeted nanoparticles exhibited a significant decrease in cell viability. Biodistribution studies of the construct, which was additionally radiolabeled with ^67^Ga, have shown excellent retention (nearly 80%) of the formulation in BALB/c mice PC-3 xenograft, even up to 24 h after direct intratumoral administration. Authors conclude their research with a remark about the possibility of customizing their Au-based system using other radioisotopes such as ^68^Ga, ^90^Y, ^177^Lu, or ^165^Er with known anticancer properties, which would improve the system efficacy.

Among Au systems tested for cancer detection and treatment, some have been developed based on PAMAM dendrimers, with gold particles embedded in the dendritic cavities. For example, Mendoza-Nava et al. effectively prepared [^177^Lu]Lu-DOTA-PAMAM dendrimers with Au nanoparticles synthesized in situ. Subsequently, they have functionalized their hybrid nanosystem with breast cancer-targeting bombesin, but also with folate, to improve the targeting efficacy ([^177^Lu]Lu-DenAuNP-folate-bombesin) [52,55]. The in vitro studies performed on the T47D breast cancer cell line, rich in both GRPR and folate receptor (FR), clearly showed specificity of the conjugates towards the receptors and presented better cellular uptake for the dendrimeric system as compared to free [^177^Lu]Lu-folate-bombesin, highlighting the beneficial effect of the carrier. Moreover, upon laser irradiation, DenAuNP-folate-bombesin led to a significant decrease in cell viability (down to 16%), thus exposing an efficient plasmonic photothermal effect. In vivo, in the athymic mice breast cancer xenograft model, [^177^Lu]Lu-DenAuNP-folate-bombesin presented satisfactory radioactivity retention up to 96 h after intratumoral injection and allowed high-quality optical imaging. A similar approach was reported later by Wang and colleagues, who also encapsulated AuNPs in the dendrimeric cavity of [^177^Lu]Lu-PAMAM-bombesin-folate conjugates and used them in the context of lung cancer [53]. Likewise, the presence of gold allowed a great enhancement of the bioimaging results, but also provided an opportunity for photothermal treatment in combination with the radiotoxicity of ^177^Lu. The researchers found selective absorption into the HEL-299 lung tumor cells overexpressing FRs and GRPRs, a greater carrier uptake than free peptides, and good retention even 96 h post-intratumoral injection.

**Figure 19:**
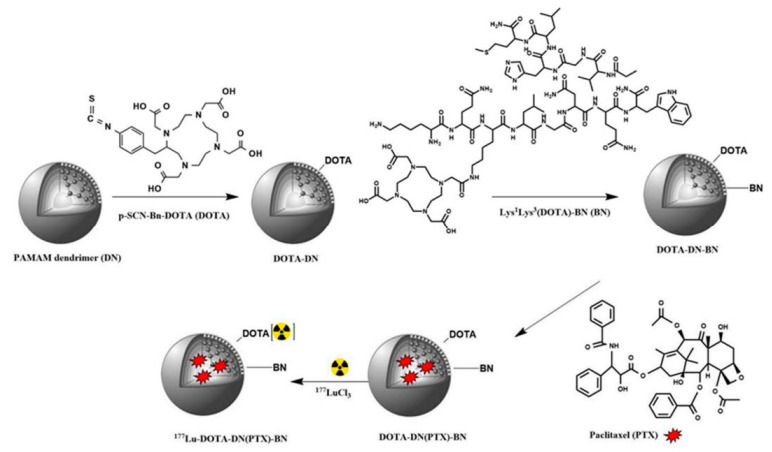
The synthetic procedures involved in the preparation of paclitaxel (PTX)-loaded PAMAM dendrimers conjugated with BBN and DOTA-^177^Lu. Reprinted from ref. [57].

Owing to their remarkable loading capacity, dendrimers were also the object of interest of Gibbens-Bandala and colleagues, who used them as carriers for a potent anticancer drug, hydrophobic paclitaxel (PTX) [57]. They exploited [^177^Lu]Lu-DOTA-PAMAM-bombesin for the theranostic delivery of PTX (Figure 19) to GRPR-positive T47D breast cancer cells. Combined radio- and chemotherapy resulted in satisfactory tumor eradication in vivo in the athymic mice xenograft model—at the 120 h mark, tumor size decreased by 15.6%. Furthermore, upon intratumoral administration, the construct is selectively bound to the receptor-bearing cells, with as much as 36.25% of the formulation retained at the tumor site and only small amounts accumulated in the pancreas and liver. Moreover, the carrier performed very well in terms of micro-SPECT/CT imaging, providing clear images of malignant tissues.

To further explore theranostic PTX delivery, Gibbens-Bandala and colleagues also evaluated PLGA nanocarriers equipped with BBN and DOTA [56]. Even simple nanoformulation of PTX outperformed free drug in in vitro cytotoxicity assessment, yet its targeted and radioactive counterpart, [^177^Lu]Lu-BBN-PLGA(PTX), led to the highest observed viability decrease. Authors postulate that PTX delivered to the cells together with radiation led to radiosensitization, and thus the synergistic effect of radio- and chemotherapy can be seen. Biodistribution studies in athymic mice with subcutaneous xenograft of MDA-MB-231 breast cancer cells have shown very promising tumor uptake despite notable liver accumulation. In the pulmonary model of the same malignancy, clear SPECT images of lesions were acquired 72 h post-injection. Most importantly, however, 8 days after administration of 3 MBq of [^177^Lu]Lu-BBN-PLGA(PTX), a more than 10-fold decrease in the tumor volume compared to the control (0.136 cm^3^ vs. 1.83 cm^3^) was found. All the results taken together confirm the theranostic potential of the proposed PTX formulation.

Targeted delivery of the antineoplastic drug together with radionuclides can be a very promising strategy. It was also exploited by the Accardo team, who, as already mentioned, intensely worked on liposomal formulations. They examined several supramolecular aggregates based on their custom-designed amphiphilic peptide derivative containing both (7–14) BBN targeting moiety and DTPA chelator [37]. Their liposomes were loaded with DOX for prostate cancer treatment and radiolabeled with ^111^In for diagnostic nuclear medicine applications. Authors have found that they are able to bind to the GRPR receptors of PC-3 prostate cancer cells efficiently and selectively. In the former case, the aggregate retention in the carcinoma after 48 h is higher than for the control, but the improvement is rather minor (2.4% ID/g vs. 1.6% ID/g), while the latter formulation provided a substantial carcinoma growth prevention (35%) compared with BBN-free liposomes, pointing to the importance of the targeting effect of the peptide. Furthermore, the DOTA-based liposomes were detectable under a clinical gamma camera, making them potentially applicable for theranostic purposes if the cytotoxicity to cancer cells was to be improved.

Chang et al. established a very different BBN-conjugated liposome system with DSPE-PEG as the base, ^188^Re radionuclide, and DOX [105]. The effects of the [^188^Re]Re-DOX-liposome-BBN intravenous injection were clearly traceable with micro-SPECT/CT imaging, which demonstrated successful targeting of the GRPR in AR42J pancreatic cancer cells and significantly reduced tumor growth rate, hence increasing the mice lifespan by as much as 86.96% compared to individuals treated with [^188^Re]Re-liposome-BBN or liposome-DOX-BBN, whose lifespans were extended by 75% and 3.61%, respectively. This is a promising result, particularly because, similarly to other nanocarriers that were not administered directly into the malignancy, some accumulation was also found in the spleen and liver.

**Figure 20:**
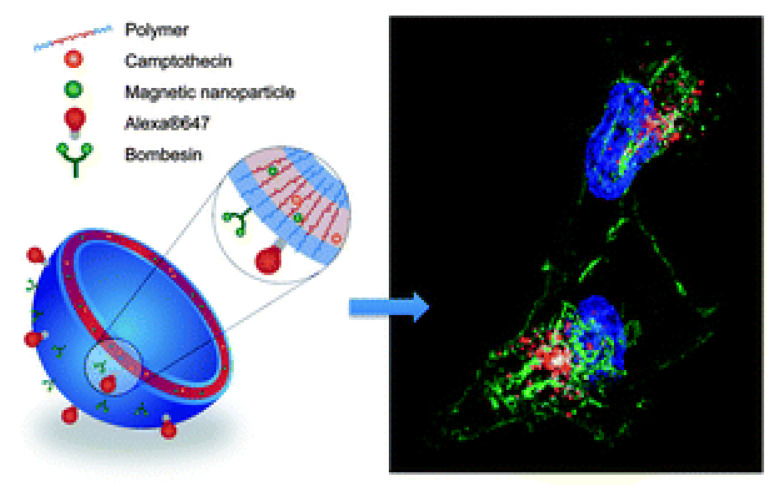
Scheme of loaded magnetic polymersomes prepared from a PEO–PPO–PEO copolymer where the hydrophobic cargo (be it a magnetic nanoparticle or drug molecule) was implemented into the PPO liposome section. Reprinted with permission from ref. [24]. Copyright 2022, Royal Society of Chemistry.

Although radioisotopes are very useful in theranostic formulations, they are not the only solution. Last but not least, hybrid nanomaterials containing IONPs may provide convenient means for diagnostic imaging—as already mentioned, they produce efficient contrast in T_2_-weighted MRI. Bleul et al. reported Pluronic^®^ L-121 delivery vehicles loaded with camptothecin (CPT) and magnetic IONPs with BBN-targeting ligands covalently bound to the surface (Figure 20) [24]. The resulting formulation exhibited anticancer properties greatly increased compared to the free drug—the MTT viability assay had reduced cell viability to around 20% compared to 40–45% for the free drug. Moreover, the applied polymer allowed for a sustained release of the drug [106]. In addition, the IONPs-containing carriers have shown very promising values of transverse relaxivity: despite the fact that loading with the CPT lowered this parameter in comparison to the drug-free nanosystem, the value was still higher than commercially available contrast agent Feridex^®^ (682 s^−1^ mM^−1^ vs. 394 s^−1^ mM^−1^ vs. 111.5 s^−1^ mM^−1^, respectively). Unfortunately, the study was not followed up with an in vivo protocol to assess the bioaccumulation in other organs or tissues and the tumor-targeting effect of these formulations.

## 7. Summary and Conclusions

Cancer continues to be a major challenge for scientists and medical professionals around the world, therefore, further development in the field of cancer detection and management remains a constant need. Oncological GRPR targeting represents a promising direction of research, from which the modern cancer management battlefield can benefit a lot. Keeping those facts in mind, this review article gives a general overview of gastrin-releasing peptide receptors, their expression, and biology in malignancies. Further, we have outlined the trends in GRPR targeting research, particularly those concerning the most frequently applied types of nanoparticles and most commonly addressed conditions. Furthermore, we have discussed the types of targeting ligands able to bind with GRPR used in nanopharmaceuticals, and the possible rationale behind the current state of the art. It suggests that the future in this field might belong to engineered peptides that confer more than just a binding motif. Moreover, we have elaborated on strategies enabling the combining of the targeting ligand with the nanoparticle carrier, both covalently and noncovalently.

Last but not least, we have reviewed many research articles reporting cutting-edge methodologies for developing advanced nanoformulations. Described strategies were designed for the treatment, diagnosis, as well as simultaneous treatment and diagnosis of various cancers. In both in vitro and in vivo preclinical animal models, most of the presented nanosystems have shown promising performance, i.e., successful targeting of GRPR-expressing cells and delivery of a variety of cargoes. However, among the numerous advantages of nanosystems, it is easy to overlook potential risks associated with the application of various nanoparticles in drug delivery, diagnostics, and theranostics.

It is important to remember that small sizes and high aspect ratio may cause the nanoparticles to interact with biomolecules (such as albumins) in an uncontrolled manner [107]. On the cellular level, there are also challenges characteristic for particular types of nanoparticles. It was found that structures with positive surface charge, such as amine- terminal PAMAM dendrimers, may disrupt cell membranes, causing mitochondria impairment and even cell lysis in a dose-dependent manner [108]. Moreover, many types of particles, particularly inorganic, such as carbon nanotubes or metal nanostructures, may stimulate excessive reactive oxygen species (ROS) formation and subsequently trigger the inflammatory response [109,110]. Nanoparticles based on metal oxides, such as titanium or selenium oxides, were shown to trigger epigenetic modifications, namely altering the DNA methylation patterns [107]. Finally, one of the issues typical for nanosystems in general, not only GRPR-targeting nanoplatforms, is accumulation in the liver and spleen in vivo. The extent to which the carrier is retained in those organs differs among the systems, however, this effect is omnipresent once the formulation is administered intravenously. Strategies such as PEGylation significantly ameliorate this issue, but do not eliminate it completely. Quantum dots, on the other hand, were shown to accumulate in lymph nodes in the mice model [107]. These facts indicate the need for further studies and should be kept in mind while developing new nanostrategies for therapeutic and diagnostic targeting of gastrin-releasing peptide receptors or any other molecular targets.

## Figures and Tables

**Table 1:** Contemporary GRPR-targeted nanosystems for cancer therapy.

Nanocarrier	Target Condition	Drug/Therapeutic Means	Reference
liposomes	prostate cancer, colon cancer, hepatic cancer	Albendazole	[78]
	lung cancer	-	[59]
	prostate cancer	Doxorubicin (DOX)	[39]
phospholipid micelles	prostate cancer	Au(III)-dithiocarbamate complex (AuL12)	[38]
polymeric micelles	glioma	Coumarin-6 Camptothecin (CPT)	[65]
	prostate cancer	Monomethyl auristatin F (MMAF)	[69]
elastin-like micelles	prostate cancer	-	[67]
		Docetaxel (DTX)	[79]
nanostructured lipid carriers	lung cancer	Doxorubicin (DOX)	[21]
solid lipid nanoparticles	breast cancer	Doxorubicin (DOX)	[63]
PLGA nanoparticles	breast cancer	Docetaxel (DTX)	[18]
	prostate cancer	Docetaxel (DTX)	[80]
gold nanoparticles	cervical cancer	RAF peptide analog (Ac-Cys-Ahx-RAF)	[73]
	prostate cancer	^99m^Tc	[27]
		^99m^Tc, ^177^Lu	[34]
		-	[81]
		Gallic acid (GA)	[51]
	prostate cancer, colon cancer	^68^Ga	[28]
gold nanorods	breast cancer	Photothermal therapy—use of near-infrared laser irradiation	[48]
	prostate cancer	Heat-labile cytotoxic free radical donor—AIPH	[82]
graphene oxide	glioblastoma	Magnetic targeting, doxorubicin (DOX), near-infrared laser irradiation	[62]

**Table 2:** Contemporary GRPR-targeted nanosystems for cancer imaging.

Nanocarrier	Target Condition	Imaging Modality	Reference
gold nanoparticles	breast cancer	X-ray imaging	[44]
	prostate cancer	CT imaging	[72]
		fluorescence imaging, CT imaging	[91]
		PET imaging, CT imaging	[92]
		microSPECT imaging, CT imaging	[23]
		microSPECT imaging, CT imaging	[22]
gold nanorods	breast cancer	PA imaging	[43]
	breast cancer, prostate cancer	-	[93]
iron oxide nanoparticles	breast cancer	MR imaging, NIR fluorescence imaging	[60]
		MR imaging, PET imaging	[54]
		PET imaging, CT imaging, MR imaging	[36]
		MR imaging	[45]
	pancreatic cancer	MR imaging	[70]
	prostate cancer	fluorescence imaging, MR imaging	[46]
			[71]
gadolinium oxide nanoparticles	prostate cancer	fluorescence imaging, MR imaging	[94]
quantum dots	-	fluorescence imaging	[74]
	prostate cancer	PET imaging, NIR fluorescence imaging	[32]
		PET imaging, CT imaging	[20]
liposomes	breast cancer	scintigraphy	[77]
	Ehrlich tumor		[75]
upconversion nanoparticles	prostate cancer	MR imaging, CT imaging, UC luminescence	[49]
graphene oxide	oral squamous cell carcinoma	fluorescence imaging	[40]
cowpea mosaic virus	prostate cancer	fluorescence imaging	[47]

**Table 3:** Contemporary GRPR-targeted nanosystems for cancer theranostics.

Nanocarrier	Target	Imaging Modality	Drug/Therapeutic Means	Reference
gold nanoparticles	prostate cancer	MR imaging (Gd) SPECT imaging (^67^Ga)	radiosensitization	[26]
		microSPECT imaging (^67^Ga)	Pt(IV) prodrug	[104]
hybrid gold nanoparticle—PAMAM dendrimers	breast cancer	optical imaging	^177^Lu radiotherapy, plasmonic photothermal therapy	[55]
	lung cancer	optical imaging	^177^Lu radiotherapy, plasmonic photothermal therapy	[53]
hybrid polymeric vesicles—iron oxide nanoparticles	prostate cancer	fluorescence imaging; potential for MRI	camptothecin delivery	[24]
liposomes	pancreatic cancer	microSPECT/CT imaging (^188^Re)	doxorubicin delivery	[105]
	prostate cancer	gamma imaging (^111^In)	doxorubicin delivery	[37]
PAMAM dendrimer	breast cancer	microSPECT imaging/CT imaging (^177^Lu)	^177^Lu radiotherapy, paclitaxel delivery	[57]
PLGA nanoparticles	breast cancer	microSPECT imaging/CT imaging (^177^Lu)	^177^Lu radiotherapy, paclitaxel delivery	[56]
